# Frontiers of the Lower Palaeolithic expansion in Europe: Tunel Wielki Cave (Poland)

**DOI:** 10.1038/s41598-022-20582-0

**Published:** 2022-09-29

**Authors:** Małgorzata Kot, Claudio Berto, Maciej T. Krajcarz, Magdalena Moskal-del Hoyo, Natalia Gryczewska, Marcin Szymanek, Adrian Marciszak, Krzysztof Stefaniak, Katarzyna Zarzecka-Szubińska, Grzegorz Lipecki, Krzysztof Wertz, Teresa Madeyska

**Affiliations:** 1 Faculty of Archaeology, University of Warsaw, Krakowskie Przedmieście 26/28, 00-927 Warsaw, Poland; 2 Institute of Geological Sciences, Polish Academy of Sciences, Twarda 51/55, 00-818 Warsaw, Poland; 3 W. Szafer Institute of Botany, Polish Academy of Sciences, Lubicz 46, 31-512 Kraków, Poland; 4 Faculty of Geology, University of Warsaw, Żwirki i Wigury 93, 02-089 Warsaw, Poland; 5 Department of Palaeozoology, Faculty of Biological Sciences, University of Wrocław, Sienkiewicza 21, 50-335 Wrocław, Poland; 6 Institute of Systematics and Evolution of Animals, Polish Academy of Sciences, Sławkowska 17, 31-016 Kraków, Poland

**Keywords:** Environmental impact, Anthropology, Archaeology, Palaeontology

## Abstract

Peopling of Central Europe by Middle Pleistocene hominids is highly debatable, mainly due to the relatively harsh climatic and environmental conditions that require cultural and anatomical adjustments. At least several archaeological sites certify human occupation in the region dated back to MIS 13-11, but they represent open-air settlements. Based on the new fieldwork conducted in Tunel Wielki Cave, we can date the human occupation traces in the cave to MIS 14-12. Bipolar-on-anvil knapping technique prevails in the lithic assemblage, made exclusively in flint. The obtained results have given ground for studying the frontiers of human oikumene and the required cultural adaptive abilities.

## Introduction

**Figure 1:**
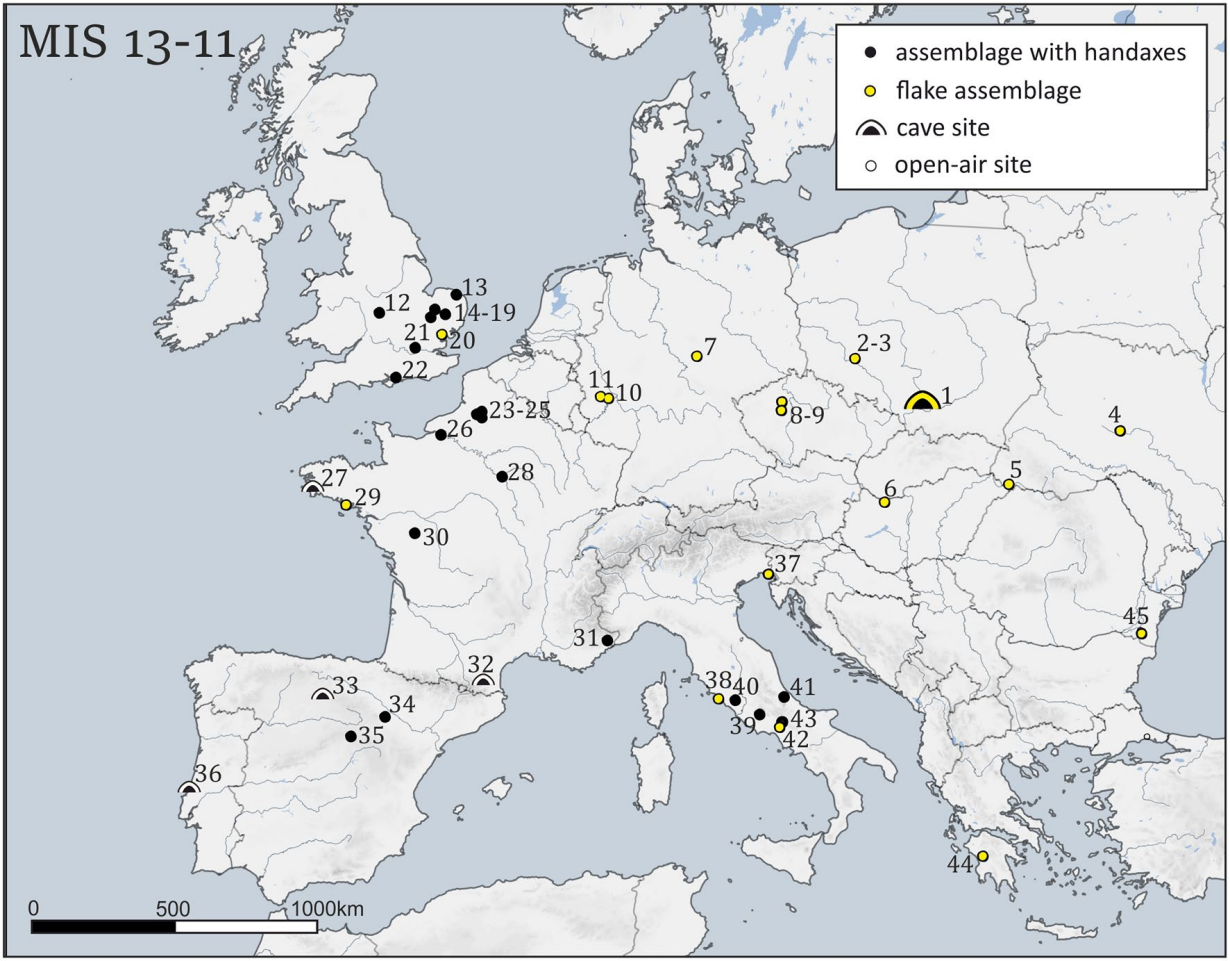
Middle Pleistocene sites dated to MIS 13-11 in Europe. 1. Tunel Wielki Cave; 2. Trzebnica 2; 3. Rusko 33, 36 & 42; 4. Medzhibozh 1; 5. Korolevo; 6. Vértesszölös 2; 7. Bilzingsleben; 8. Račinĕves 9. Karlštejn-Altán; 10. Miesenheim I; 11. Kärlich-Seeufer; 12. Waverly Wood; 13. Happisburgh Site 1; 14. Warren Hill; 15. High Lodge; 16. Beeches Pit; 17. Hoxne;18. Elveden; 19. Barnham; 20. Clacton-on-Sea; 21. Swanscombe; 22. Boxgrove; 23. Cagny-la-Garenne; 24. Ferme de l’Epinette; 25. “Rue de Cagny”; 26. Saint-Pierre-les-Elbeuf; 27. Menez Dregan; 28. La Celle; 29. St. Colomban; 30. La Grande Vallé; 31. Terra Amata; 32. Arago; 33. Atapuerca Galeria; 34. Aridos; 35. Ambrona; 36. Gruta da Aroeira; 37. Visogliano 38. Ficoncella; 39. Fontana Ranuccio; 40. Castel di Guido; 41. Valle Giumentina; 42. Isernia-la-Pineta; 43. Guado San Nicola; 44. Marathousa; 45. Dealul Guran (All site details and references in Supplement Table 1). Map generated using QGIS v. 2.14 (qgis.org), digital elevation model modified from STRM^37^ raster data, boundaries and river layers modified from Natural Earth (naturalearthdata.com) vector data.

The first peopling of Europe started from its southernmost part, although the potential migration routes are still under debate^1–6^. The inhabited regions resembled the north African and Near Eastern climatic and ecological conditions, so the hominin migration could be a part of the faunal renewal of the region^7–11^. The human expansion further to the north, out of the Mediterranean niche, required severe cultural and physical adaptations^12^. Even though all ephemeral archaeological evidence has been discussed, traces of Middle Pleistocene human occupation in Central Europe, predominantly north of the Carpathians, are extremely scarce and late compared to the other regions of Europe. It is primarily due to the geological history of the European Lowlands^13^ and paleoclimatic conditions, which prevented hominids from crossing the Carpathians. Well-recognized Lower Palaeolithic open-air sites to the south of the Carpathians such as Vértesszölös, Korolevo (level VI)^14–17^ have only scarce analogies on the northern side of the mountains in Poland, e.g., Trzebnica 2, Rusko 33, 36 and 42^18,19^. However, the Middle Pleistocene chronology of the Trzebnica assemblage has been recently questioned^20^. Still, together with several open-air sites found in central Germany (Bilzingsleben, Schöningen 13, Kärlich-Seeufer, Mauer)^21–28^, they mark a frontier of the human expansion which took place during MIS 13-11 (Fig. 1).

Interestingly, all the known Lower Palaeolithic sites in Central Europe are open-air sites. It is because there are only a few cave sites in the region with preserved Early and Middle Pleistocene sediments^29,30^. In the largest karstic region situated north of the Carpathians, i.e. the Kraków-Częstochowa Upland, with over 2000 caves, only a few sites with Middle Pleistocene sediments have been identified so far^31^. One is the well-known Biśnik Cave, with the lowest strata containing stone artefacts (19a–d) dated previously up to MIS 8/7 or even MIS 9^32–34^. The most recent analysis of the unusually abundant fossil material confirms an age no older than MIS 10 in the lowermost layers of Biśnik Cave^35^.

The scarcity of Early and Middle Pleistocene cave sediments is typical of caves in Kraków-Częstochowa Upland. The reasons for this phenomenon are unknown; however, we expect this is mainly due to the washing out of the sediments during several Middle Pleistocene glaciations when Kraków-Częstochowa Upland was surrounded by ice sheet in the form of a concave nunatak^30,36^. In such a context, traces of human occupation found in Tunel Wielki Cave in the southern part of Kraków-Częstochowa Upland are unique and highly significant. We believe that they can shed more light on the distribution of genus *Homo* in Europe and on hominid life on the frontiers of their ecumene and adaptive abilities.

### Tunel Wielki Cave

**Figure 2:**
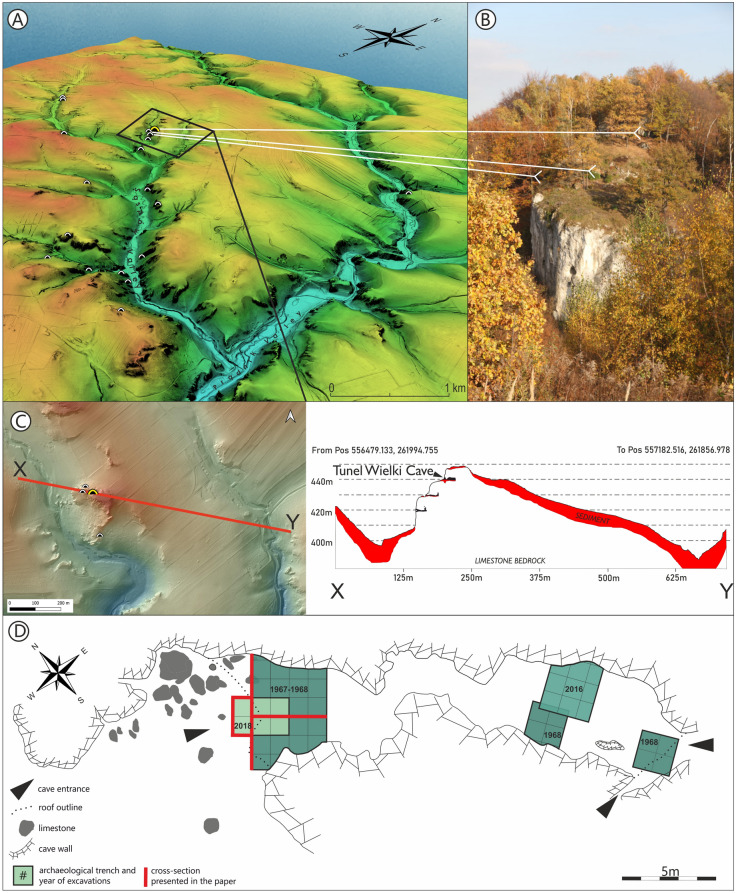
Location of Tunel Wielki Cave. A. Sadlane rocks with entrances of Tunel Wielki Cave on the top and two other archaeological sites 'Nad Niedostępną' and 'Pod Tunelem' Rockshelters located underneath. B. LiDAR map of a karstic region of the Sąspów and Prądnik valleys with location of Tunel Wielki Cave and other cave archaeological sites. C. Section through the Sadlane rocks. D. Plan of Tunel Wielki Cave with the exact location of the archaeological trenches (plan based on Madeyska^41^ and 1967/68 fieldwork documentation).

Tunel Wielki Cave is located in the karstic region of Kraków-Częstochowa Upland, ca. 20 km north of Kraków. The site is the uppermost (440 m a.s.l.) of several cavities located in *Sadlane* rocks in Koziarnia Gorge (Sąspów Valley) (Fig. 2). The cave has the form of a 24 m long tunnel with two openings heading north-west and south. It consists of two chambers connected by an eight metres long narrow corridor. The spacious south chamber has a high ceiling, but recently, a huge boulder almost entirely covered its opening. The northern chamber of much smaller size has a large entrance of 6 × 1.5 m. The south entrance overlooks a steep slope, whereas a flat terrace sits in front of the north one.

The cave was excavated in 1967–1968 by W. Chmielewski, who opened four trenches placed both in the north and south chambers (Fig. 2)^38^. The south chamber was revisited in 2016. Both excavations revealed the presence of solely Holocene and Final Pleistocene strata of up to 1.5 m thickness^39,40^.

**Figure 3:**
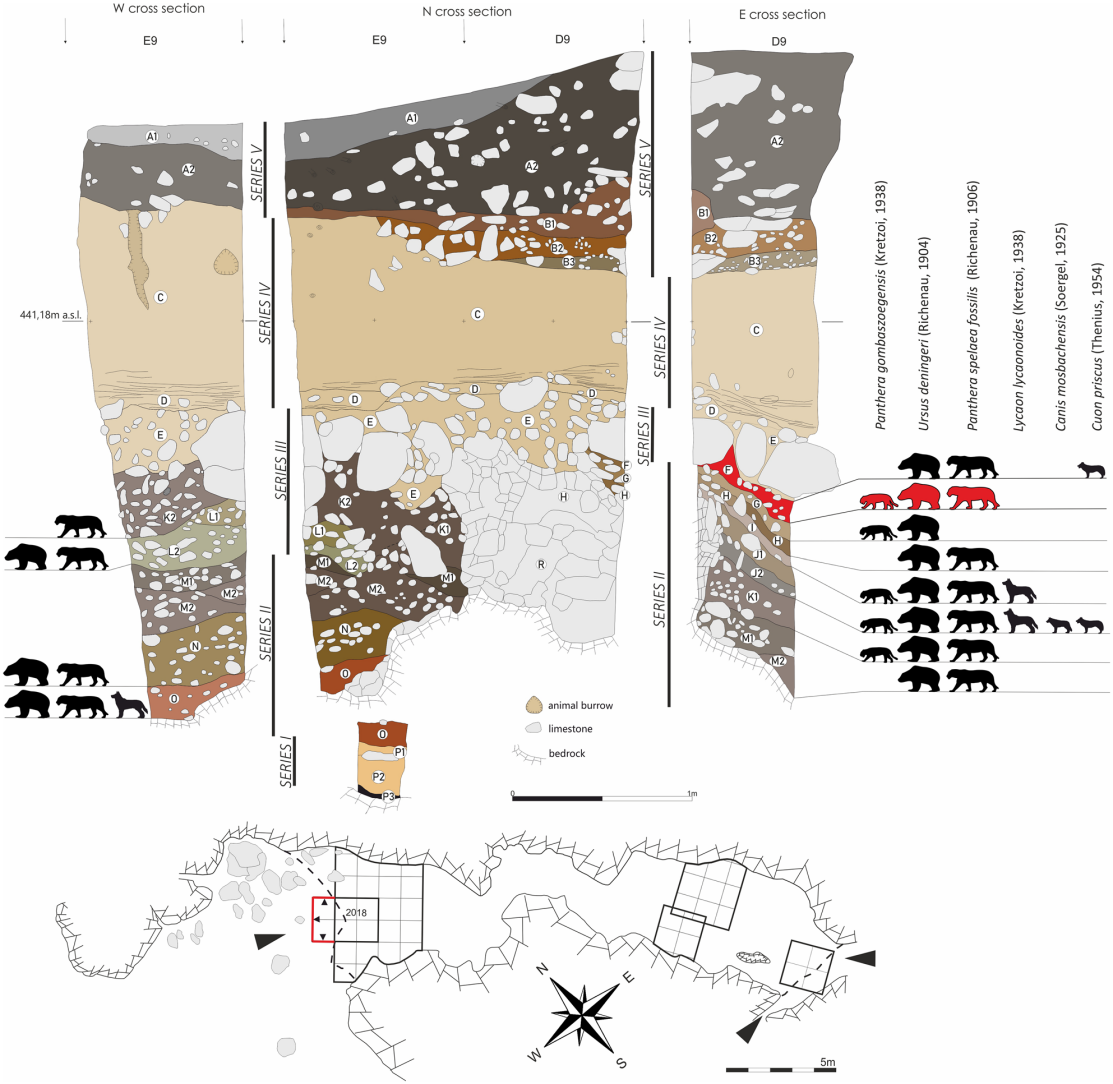
Cross section of 2018 fieldwork in Tunel Wielki Cave and distribution of carnivores which are chronological markers within different Pleistocene layers. Layer F is marked in red.

The stratigraphic sequence found in the north chamber is of interest here (Supplement Fig. S2 and Fig. 3). Chmielewski uncovered a 4.5 m deep sequence, subdividing it into 15 litho-stratigraphic units^41,42^ including Pleistocene loams covered with a ca. 1 m thick loess layer and a 1 m thick Holocene humic strata^41^. Already then, *Pliomys lenki* (=*Pliomys coronensis*) was recorded among multiple faunal remains found in the lower loam layers^43,44^. At that time, it was interpreted as a possible secondary re-deposition of Middle Pleistocene materials within the site. Archaeological artefacts found in the upper part of the series of loamy strata were therefore identified as late Middle Palaeolithic^41^. All excavated bird remains were attributed to the Holocene^45,46^.

New fieldwork in 2018 aimed to test the previous hypotheses. The first goal was to verify the presence of Middle Pleistocene layers at the site, while the second one was to determine the chronology of the stone artefact assemblage found within the loamy strata. We reopened the old trenches in the north chamber and extended them to collect geological, chronostratigraphic and paleoenvironmental data. The analysis of the small mammal assemblage clearly indicated that, indeed, the cave sediments contained fauna belonging to two horizons^47^. The uppermost assemblage, related to the Late Pleistocene and the Holocene, was composed of a few remains of *Dicrostonyx torquatus* and *Lemmus lemmus* in the loess layers C and D, with a typical Holocene fauna in the upper layers A and B. The lower assemblage found within the loamy strata (layers F-P) is composed of a different list of rodent taxa, which were attributed, among others, to the following species: *Arvicola mosbachensis*, a small *Alexandromys oeconomus*, *Allocricetus bursae*, *Microtus* (*Terricola*) aff. *arvalidens*, *Pliomys coronensis (*= *Pliomys lenki)*, and *Pliomys episcopalis*. Such a taxonomic composition allowed us to correlate this part of the small mammal sequence with the Early Toringian. However, the faunal remains of the lower assemblage probably accumulated in two different intervals, related to interstadial and stadial environment conditions, although the second one was more doubtful. Thus, the accumulation of these layers took place between MIS 14 and MIS 11^47^.

The analyses of the large mammals, primarily carnivores, collected during both the old and new fieldwork also indicated the presence of Middle Pleistocene species, e.g. *Panthera gombaszoegensis, Canis mosbachensis, Ursus deningeri,* in the lower part of the section (layers 1–11 according to Madeyska, 1988, see also Supplement Fig. S2)^48,49^. Therefore, the preservation of Middle Pleistocene layers in the Tunel Wielki Cave was verified thanks to the faunal assemblage. The remaining question was if the human occupation traces found at the site in the loamy layers could be of the same chronology. This paper aims to answer this question.

## Stratigraphy of sediments

The stratigraphy of Tunel Wielki Cave is composed of five distinct lithological series. In the uppermost series V and IV, the sequence is analogous to other cave sites in the region. The uppermost part of the section, i.e., series V, consists of 0.8–1.0 m thick blackish and brownish humic silts with limestone debris accumulated during the Holocene (Supplement Table S3- layers A2, B1, B2 and B3). Series IV includes silty strata C and D of ca 1.0–1.1 m thickness. The deposition of this series was a type of simultaneous loess and limestone debris accumulation (layer D), followed by washing and loess accumulation (layer C). Series IV has lithostratigraphic analogies in other cave sites in the region, indicating its MIS 2 chronology^42^. We confirmed the Last Glacial Maximum chronology of the loess stratum using OSL dating. A single OSL sample collected from the upper part of layer C gave a date of around 23–24 ka (Supplement Fig. S4 and Supplement Methods S5).

The underlying series of sediments do not have direct analogies in the region. Series III consists of layers with mixed silty loam and silts and large limestone clasts (layers E, K2, L1 and L2). These strata are of inhomogeneous texture and contain numerous ripped-up clasts of silty sediments within a loamy matrix. They fill an erosional structure (a channel) visible in the western part of the transversal cross-section (Fig. 3). Thus, there is a clear unconformity between Series III and II. The underlying Series II contains loams with abundant nodules of manganese precipitates and limestone debris (layers F, G, H, I, J1, J2, M1, M2, N, O and R). These layers of variable colouration contain a significant amount of blackish, heavily petrified animal remains and isolated flint artefacts. Sediments of Series I fill the depressions in the bedrock. This series consists of silty and sandy sediments without limestone debris (layers P1–P3).

U-series dating of the enamel from a single cave bear (*Ursus deningeri*) canine tooth from layer M revealed its age to be 48.3 ± 0.3 ka BP (Supplement Table S6). The specimen was also sent for collagen extraction to check the results with radiocarbon dating. Unfortunately, the specimen contained no collagen.

## Sedimentology

Layer F was the uppermost loamy layer containing stone artefacts; therefore, the analyses focused on answering a question about the depositional processes taking place above, below, and within layer F. The presence of large limestone blocks limits the readability of the layer's boundaries. Layer F, together with lower sedimentary units (layers G down to K), shows distinct inclination (around 30° towards E and 30–40° towards S). However, the analysis of the spatial distribution of the aforementioned layers reveals that this inclination has a limited range. The cross-section generated by W. Chmielewski and T. Madeyska^41^ documents a U-shaped bending of these layers, which more-or-less reproduces the concave morphology of the bedrock. Such a layout may be interpreted as postdepositional plastic deformation likely related to subsidence.

Layer F exhibits typical micromorphological features of sediments deposited in the low-energy environment of a cave floor: massive structure, silty clay grain size composition and the presence of numerous bone and tooth fragments, which correlates with data from other sites^50–52^. It is also characterized by periglacial or frost-action-related micro-structures (see Supplement Data S7). The inclination of micro-features suggests that the entire sediment package has been re-oriented by the subsidence. The survival of voids, stratigraphic unit boundaries and other micro-features indicates that the reorientation process involved the whole package of now-inclined layers without disturbing their inner structure. Similar inclined planar voids also occur within layer G and weakly developed ones in layer H (Supplement Fig. S8: e–h), which indicates that the frost-action event affected the entire package of layers F–G–H.

The original depositional processes cannot be exactly characterized for layer F, as most of the original sedimentary features were disturbed by later frost action and subsidence. However, it is clear that the material of layer F is different from the sediments of underlying layers G and H (see Supplement Table S9). The material of layer F can be therefore regarded as the result of a new depositional event and supply of fresh clastic material, and not an important re-deposition of older sediments. The upper boundary of layer F is erosional. It is covered by layer E, which is characterized by several features indicating its colluvial origin: i) unconformity at the bottom; ii) erosional channels located at the bottom; iii) a complex macroscopic texture with clasts of compacted sediments chaotically dispersed within a loose matrix; iv) a loose microscopic structure with packing voids (Supplement Fig. S8: a, b); and v) presence of numerous clay balls, some of them containing bone fragments (Supplement Fig. S8: a, b). A hiatus between layers F and E was possibly filled by other layers of Series III (layers K2, L1 and L2), present in another part of the site. Layer K2, also analysed micromorphologically, exhibits similar features to the ones observed for layer E (Supplement Fig. S8: c,d).

## Lithic assemblage

The artefacts were made solely from the local Jurassic flint. Chert nodules do not occur naturally in caves in the region. Although chert occurs here in limestones, these represent other limestone facies (deep-water bedded limestones with marls and olistoliths) than the facies where the caves developed (massive limestone from carbonate build-ups)^53^. The nearest outcrops of the raw material can currently be found in the bottom of the gorge, ca. 50 m below the cave, and along the edge of the Sąspów Valley in the secondary clay deposits.

The artefacts are heavily patinated, and their edges show a high degree of postdepositional damage. Almost all the edges are either postdepositionally retouched or abraded. A single artefact was burned. All the artefacts have shiny surfaces. Postdepositional damage can be found in the MIS 3 and older flint assemblages in the region (see Koziarnia Cave Middle Palaeolithic assemblage for a comparison^54^). The specificity of postdepositional pseudo-retouches was recently studied by Cyrek and Sudoł^55^ in Biśnik Cave, another Middle and Upper Pleistocene cave site in the region. Quite a similar effect was also observed for bones in Biśnik Cave. There, it was explained rather by tension due to frost weathering than compression^50^, although the main factor was still frost processes. The edge damage is believed to be an effect of tension due to pressure from swelling and shrinking sediments within a periglacial environment^55^.

**Figure 4:**
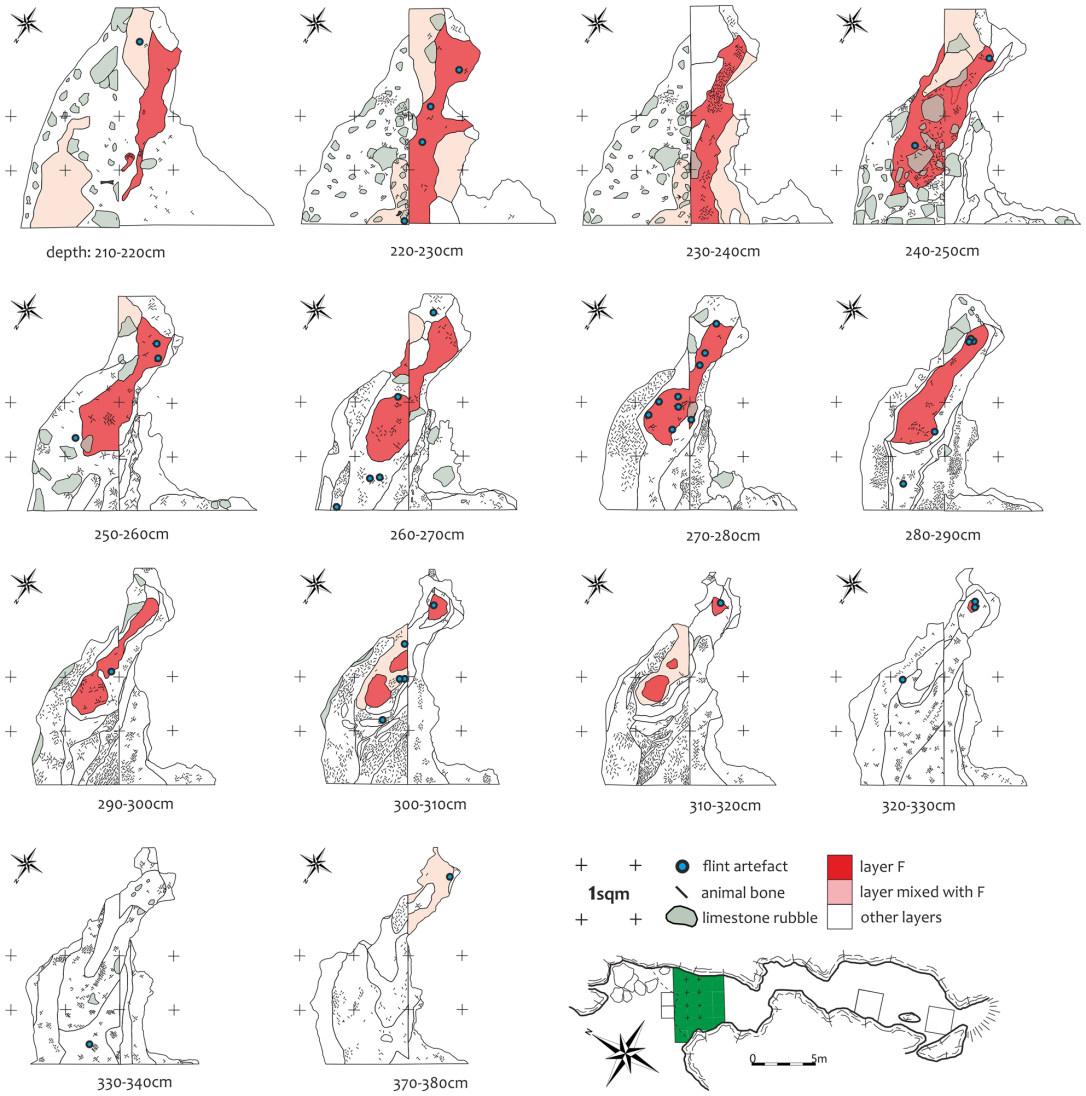
Tunel Wielki Cave. Planigraphy of stone artefacts at different depths under the surface. Layer F is the uppermost one of the whole package of Pleistocene loams. It was preserved in the form of an inclined lens surrounded by the underlying strata, which were found in the form of circles around layer F. The stratigraphic position of the layer and the artefacts was caused by creeping processes leading to the slow movement of the whole loam package toward the vertical chimney in the bottom of the bedrock.

Due to the uneven distribution of artefacts in both trenches (trench 1967–68 of 20 m^2^—43 pieces; trench 2018 of 2 m^2^—118 pieces), we cannot speculate about the spatial distribution of artefacts. Moreover, the loamy sediments, including layer F, show traces of slow sediment creeping towards the vertical chimney at the end of the chamber. Layer F was preserved in the form of an inclined lens of sediment, in the plan-view, surrounded by the originally underlying layers (Fig. 4). The lens could be found at a depth between 210 and 330 cm under the surface. The very bottom parts of layer F were found just above the chimney. A concentration of three cores was found at this place (Fig. 4: 320–330 cm). The highest concentration of large debitage was found at a depth of 270–280 cm under the surface (Fig. 4: 270–280 cm).

**Figure 5:**
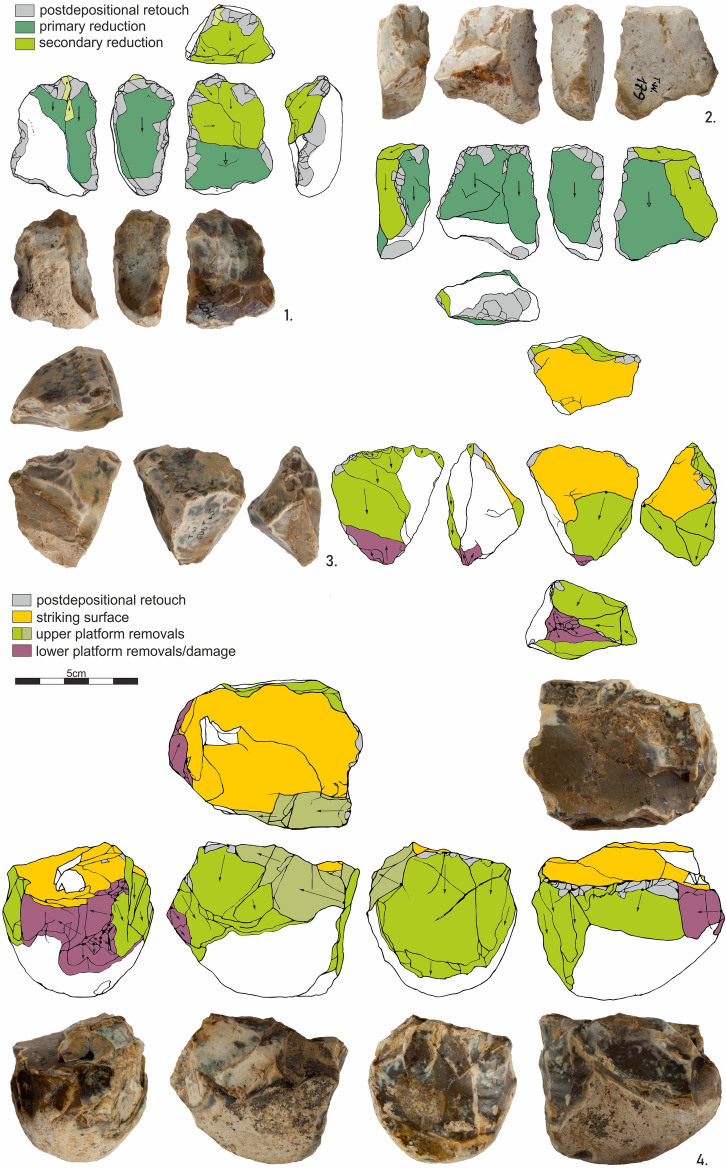
Tunel Wielki Cave. 1–2. Cores on flakes; 3–4. Cores made on flint nodules, with visible lower platform removals and crushes.

The assemblage consist of ten cores or their fragments. Four cores were made from nodules. Another four were made on flakes. (Fig. 5). Except for a single piece, they all are small in size, not exceeding 50 mm in length, and triangular in cross-section. The cores on flakes were made on thick flakes. They were exploited on their ventral side, with a butt treated as a striking platform. The distal edge of the core/flake is crushed in four cases, with traces of removals spreading onto both sides of the core. A single large core with a changed orientation was made on a cortical round flint nodule. After reorientation, its primary flaking surface was used as a striking platform for further core exploitation. In both knapping positions, the surface opposite to the striking platform is crushed or the edge has traces of removals detached simultaneously on both sides of the core (Fig. 5).

In both core types, the knapping scheme is quite similar. Flakes were detached from the sides of the core. Due to the morphology of the cores, the obtained flakes were asymmetric, with a single sharp and second slightly blunted or cortical edge. In a single case, the core-on-flake was exploited transversally (Fig. 5:1).

**Figure 6:**
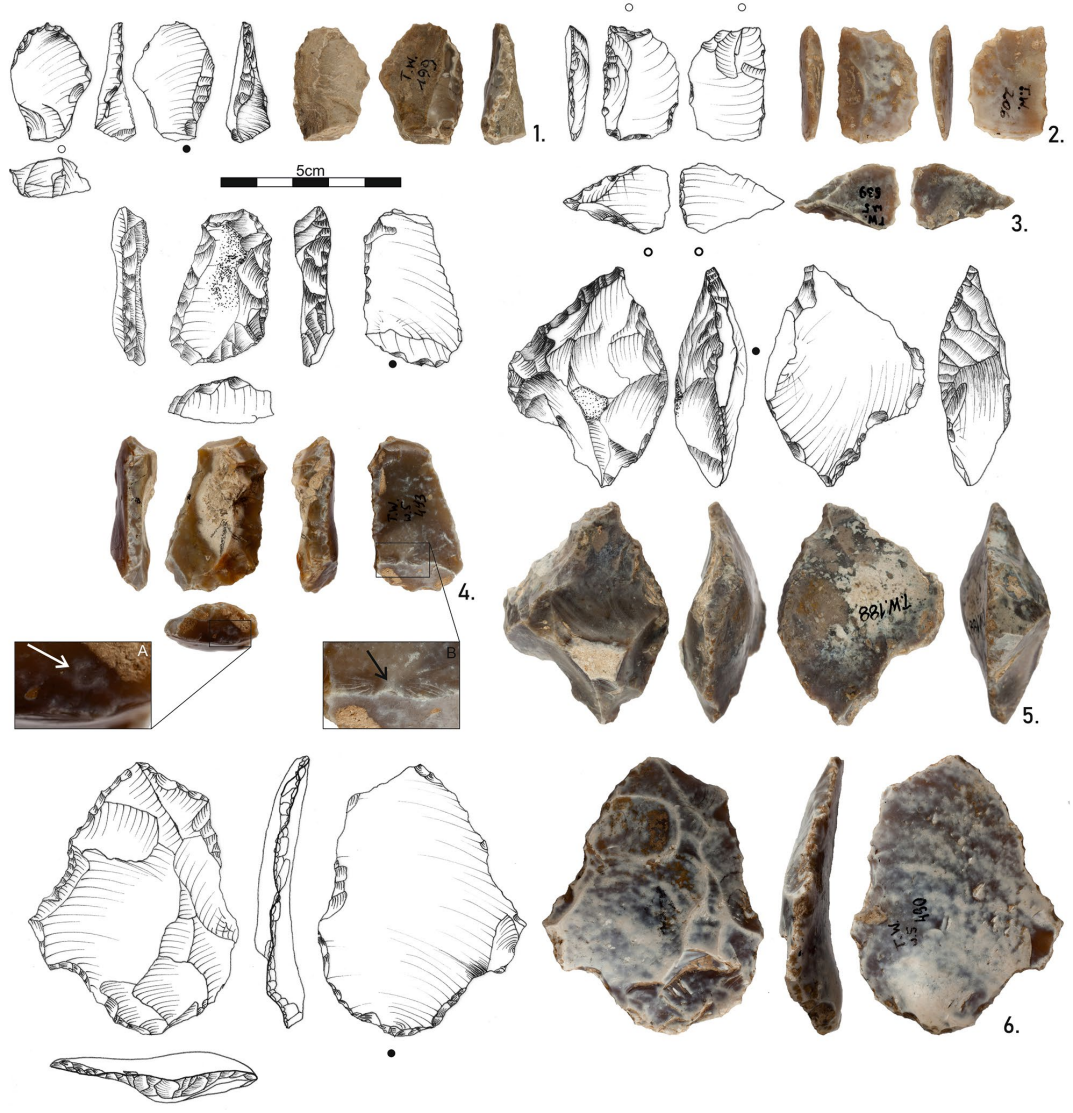
Lithic tools from Tunel Wielki Cave. 1. Kombewa flake; 2. Kombewa flake with a retouched edge; 3. Broken Kombewa flake; 4. Longitudinally retouched flake; 5. Transversally retouched flake; 6. Flake with heavily postdepositionally damaged edges. Its big size, longitudinal curvature, massive bulb and equal thickness throughout its whole length indicate the use of free-hand technology; A. Hertzian cone of the unsuccessful flake detachments; B. Protruding point of percussion on the ventral side of the flake.

The debitage consists mainly of thick irregular backed flakes of unidirectional or orthogonal scar pattern, but bidirectional removals also appear (Supplement Fig. S10). Typo-technological attributes of artefacts can be found in Supplement Table S11. Cortical flakes consist of 50% of the debitage. The cortex is located mostly lateral right. Three Kombewa flakes were identified in the assemblage (Fig. 6). Flakes have big convex or flat butts, either prepared with a single removal or left cortical without preparation. The point of percussion can be seen on the ventral side. In seven cases, it has a form of half of the Hertzian cone (Fig. 6:4). Bulbar scars were identified in 8 cases. Hertzian cones of the unsuccessful percussion attempts can also be identified on the butts (Fig. 6:4). Besides the protruding fracture point, flakes have no bulbs (except two artefacts with big bulbs). Intensively curved ripples or even compression rings can be observed on the ventral sides of the flakes. Radial fissures are well visible, especially near the point of percussion. A specific feature of a split of the ventral surface in its distal part can be observed in several pieces.

**Figure 7:**
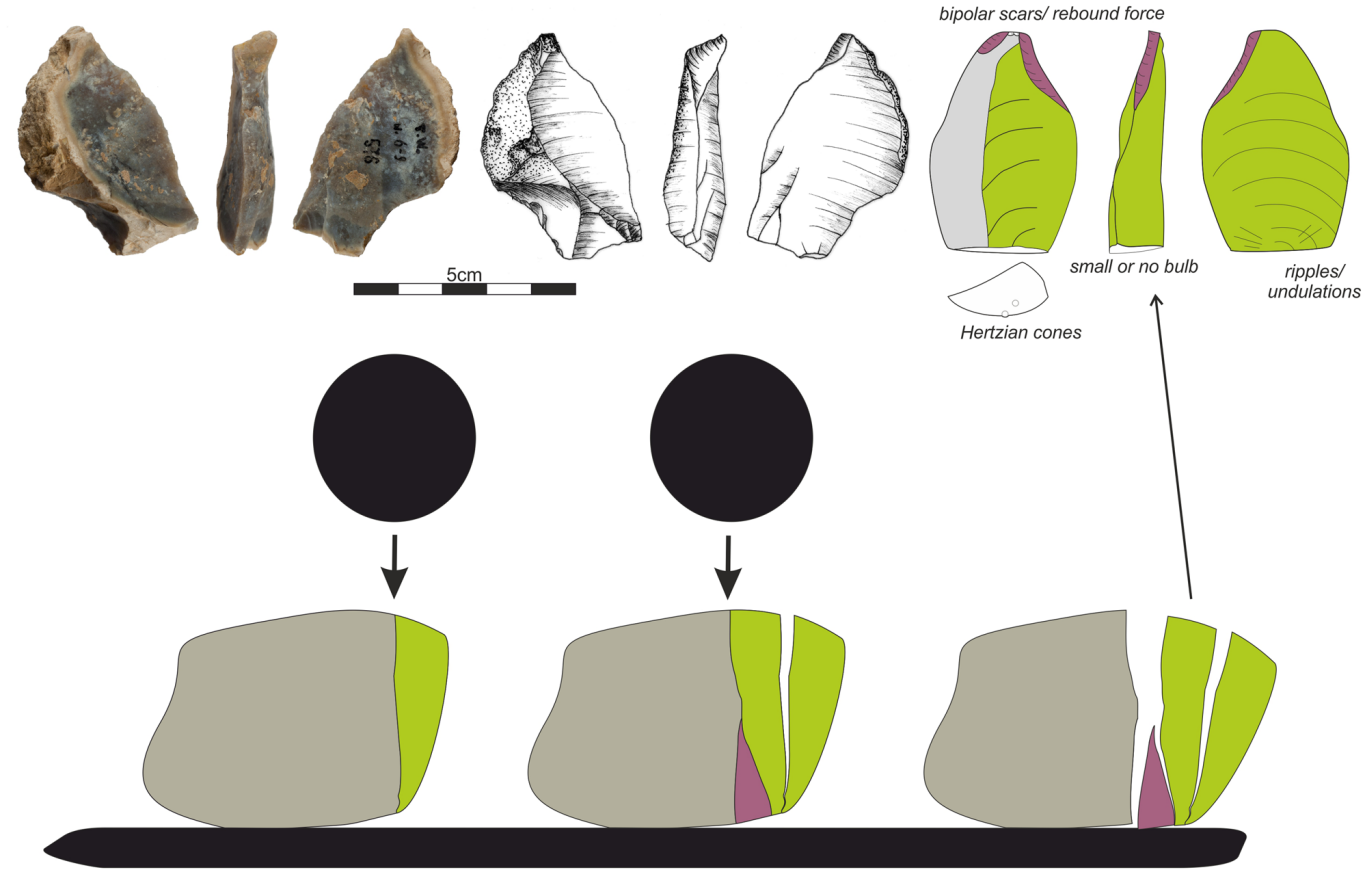
Bipolar-on-anvil reduction scheme and the technological features which might be observed on debitage.

The technological features of the debitage fit the knapping scheme identified in cores. Taking into consideration the following features, we assume that one of the knapping technologies used was the bipolar-on-anvil technique (Fig. 7):

- damage visible on the opposite end of the cores,
- second striking platform with removals or crushing detached on both sides of the core,
- presence of multiplicated, heavily curved ripples or compression rings,
- lack of bulb,
- specific split of the ventral surfaces of flakes.

The features presented here fit the general characteristics of the bipolar-on-anvil debitage obtained during experimental studies^52,53^. However, the presence of big butts^56,57^ (e.g. Figure 6:4) indicate that the free-hand technique was also used. We cannot rule out that only some cores, due to their small size, were placed on a solid surface simply to stabilize the nodule, while the majority of knapping was done free-hand.

Only one single large flake shows distinct technological features (Fig. 6:6). It is a slightly curved piece with a massive bulb and equal thickness along the main axis. Its size and technological features do not fit the cores in the assemblage. We assume the flake was detached using the free-hand techniquefrom a core of at least 10 cm in diameter.

Due to the severe postdepositional damage to the edges, the identification of the intentional retouches and tools was impeded Three flakes were identified as intentionally retouched pieces due to continuous edge retouch, which changed the edge parameters in the same manner. One of the tools was made on a Kombewa flake, retouched on the longitudinal edge with a semi-flat far-reaching retouch (Fig. 6:1). The second flake was also longitudinally retouched on either one or two edges (Fig. 6:4). The transversal semi-steep retouch of the third flake created a protruding part of the perforator's tip type (Fig. 6:5).

## Results of the wood charcoal analysis

The results of the anthracological analysis are based on charcoal fragments found in lithological Series II and III: in the first, there are charcoal fragments that were obtained from 10 samples coming from 7 layers, while in the latter, charcoal fragments that appeared in 7 samples were included. In all layers, they co-occurred with lithic artefacts (Table 1). The wood charcoal assemblage documented in layers of the lithological Series II was very small and only contained 13 charcoal fragments of relatively small size (no bigger than 3 mm in the longest dimension). They represent six taxa identified to genus level (*Corylus*, *Fagus*, *Quercus*, *Picea* or *Larix*, *Salix*, *Salix* or *Populus*). Three charcoal fragments were badly preserved and were determined as conifers. Only one charcoal fragment appeared in layer F, which is related to the highest accumulation of lithic artefacts (Table 1). It belonged to beech *Fagus* and may indicate mild climatic conditions, mostly associated with interstadial environmental conditions. Charcoal fragments of *Corylus* and *Quercus*, found in layers J1, R and M, may also suggest rather warm climatic conditions. The remaining taxa could have occurred both during stadials and interstadials. Unfortunately, from the region of Kraków-Częstochowa Upland, no pollen data are currently available for the period representing MIS 14-12, but a succession of vegetation of the Ferdynandovian interglacial (MIS 15-13) and part of Sanian 2 (MIS 12)^58,59^ is known thanks to the palynological sequences from eastern Poland. In the lithological Series III layers, 15 charcoal fragments appeared belonging to six taxa (*Betula*, *Corylus*, *Fagus*, *Fraxinus*, *Quercus* and *Picea* or *Larix*).

**Table 1:** Results of wood charcoal analysis from lithological Series II and III from Tunel Wielki Cave.

Bag N° inventory	Trench	Metre	Layer	Depth	Occurrence of lithics within the layer	*Betula*	*Corylus*	*Fagus*	*Fraxinus*	*Picea/Larix*	*Quercus*	*Salix*	*Salix/Populus*	coniferous	broad-leaved
**Lithological Series III**															
TWB736	2	E9	E	180–190	+									1	1
TWB750	2	D9	E	200–220	+		1								
TWB756	2	E9	E	200–220	+					1				1	
TWB789	2	E9	K2	230–240	+ +	1			1	4					
TWB790	2	E9	K2	230–240	+ +									2	
TWB796	2	E9	K2	230–240	+ +						1				
TWB803	2	E9	K2	240–260	+ +			1							
**Lithological Series II**															
TWB749	2	D9	F	200–220	+ + + *			1							
TWB769	2	D9	G	200–220	+									1	
TWB770	2	E9	G	220–240	+					1					
TWB762	2	E9	G	200–220	+								1		
TWB758	2	E9	G	200–220	+					1					
TWB793	2	D9	I	230–240	+					1					
TWB783	2	E9	J1	220–240	+		1								
TWB829	2	D9	R	240–260	+						1				
TWB876	2	E9	M2	300–320	+		1				1	1		1	
TWB915	2	E9	O	360–380	+									1	

*The burned flake.

## Discussion

Although the solifluction of material (or a part of it) into a cave can never be entirely ruled out, especially in the case of sediments that have undergone secondary disturbances, in the case of layer F, we can observe several indications of an internal cave deposition:

(i) numerous bone and teeth fragments visible through micromorphology, which is typical for carnivore dens, typically in caves and rockshelters^60–63^;

(ii) this type of sediment, with a macroscopic appearance of brownish or greyish clayey loams with limestone debris and bones, is quite typical for the Middle Pleistocene and lower Upper Pleistocene series of cave deposits in the region (e.g., in Biśnik Cave, Ciemna Cave, Żarska Cave^64,65^) and have never been found outside of caves.

(iii) the sediment exhibits very poor sorting (clay, silt and larger clasts of bones and limestone together) and lack of lamination. This excludes a transportation that could have affected grain size composition and material integrity (only transport of a whole package is acceptable here). Therefore, lithics within the sediment can be regarded as contemporary to the other material and can be dated by this material (e.g., by fossils).

(iv) the comparison with other sediments at the site, i.e., Series III, reveal a lack of sediment movement features, identified both macroscopically (lamination, variable litho-types intercalated) and microscopically (clay balls, loose packing).

(v) Tunel Wielki Cave is located high in the valley on the top of the ridge between two gorges. It is also the uppermost cave within the whole cave system in the Sadlane Rock. The section of the rock can be seen in Fig. 2C. There is a small plateau above the cave surrounded by subvertical cliffs, thus there is no space above the cave for such a volume of sediments and bone accumulations that could have been redeposited later into the cave. Any slope movements were possible only from the cave but not into it.

Nevertheless, even if these sediments were somehow moved into the cave, this would imply that they were of even earlier date than that indicated for their final deposition. Notwithstanding this, the fossil fauna allows the chronology of the layers to be established regardless of the deposition modes involved.

We assume that layer F represents the original depositional context for this assemblage due to the following observations: (i) the similar state of preservation of all the artefacts found in the Pleistocene loams (series II); (ii) no typo-technological differences between artefacts; and (iii) most importantly, traces of postdepositional movement of the sediments, which might have caused a change in the original position of the artefacts. However, we cannot exclude that some of the artefacts found in strata located below layer F represent separate human occupation episodes. We cannot also conclude about the contemporaneity of the assemblage found in layer F. It can represent single as well as multiple settlement episodes at the site. Nevertheless, the faunal composition of layer F and the underlying strata indicate their Middle Pleistocene chronology.

Unfortunately, the small mammals of layer F are very few (NISP = 8); only *Dicrostonyx* sp., *Alexandromys oeconomus* and *Lasiopodomys* (*Stenocranius*) ex. gr. *gregalis* are attested. This layer was related to the Middle Pleistocene by its stratigraphical position; nonetheless, the small dimensions of *Alexandromys oeconomus* might also weakly support this chronological attribution^47^. Molluscs and bird species can give no grounds to determine the chronology of the strata (Supplement Data S14 and S15).

The large mammals, especially the carnivores, are the best chronological markers within the analysed proxies. The lack of human intervention traces on bones found in Series III, II and I (including cutmarks), together with a predominance of carnivore remains in these layers, may indicate that the cave was used mainly as a carnivore den.

Among the carnivores, the immense *Lycaon lycaonoides* is present, the last occurrences of this species are recorded in Hungary and Poland around MIS 11^16,35,66^. Co-occurring was the Mosbach wolf *Canis mosbachensis*, with the presence of individuals slightly larger and more robust than those known from European sites dated to MIS 15-12, such as Château Breccia, Hundsheim, Jockgrim, Mauer, Mosbach 2, Południowa Cave or Urd Cave. This slow increase in body size, very characteristic for the period MIS 12-11, was correlated with the decline and reduction of the competition pressure of lycaon^35^. Both species were found in layer J; additionally, *Lycaon lycaonoides* is present in layers I and O (Supplement Table S16 & Fig. 3).

**Figure 8:**
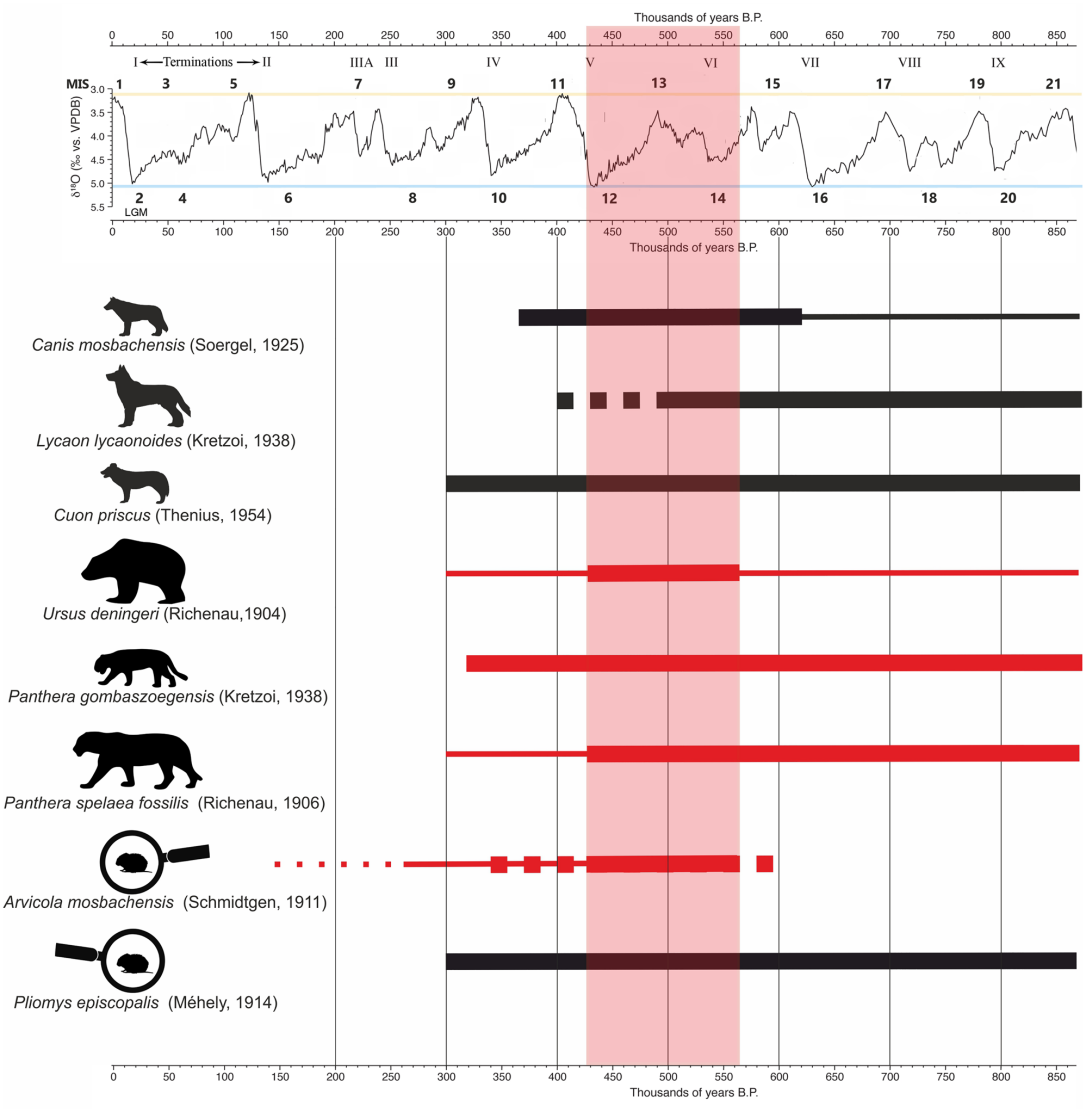
Reconstructed chronology of the carnivores and small mammals from Tunel Wielki Cave based on their morphology. The thin lines refer to the occurrence of the species in Central Europe. Thick lines refer to the chronology of the individuals of the morphology observed in Tunel Wielki Cave. The dotted line refers to the probable occurrence. The red bar indicated the most probable chronology of the human occupation in Tunel Wielki Cave based on mammals' taxonomy and morphology.

The Eurasian jaguar *Panthera gombaszoegensis gombaszoegensis* (the pantherinae cat) was found in layers F, G, I, J2 and K1 (Fig. 3). This species finally went extinct during MIS 10-9 ^48,67–69^. The detailed analysis of the most abundant species, *Ursus deningeri*, which is present in all the layers of Series II, showed strong similarity to European sites dated to MIS 15-12, such as Château Breccia, Hundsheim, Jockgrim, Mauer, Mosbach 2, Południowa Cave or Urd Cave. Among them, the most useful morphodynamic index P4/p4 positioned *U*. *deningeri* from Tunel Wielki Cave among those sites (Fig. 8). Remains of this ursid from this locality showed a lower evolutionary stage when compared to sites dated to MIS 11-10, such as Swanscombe, the complex of layers 19a-d from Biśnik Cave, Draby 3, or Za Hájovnou Cave^49,70,71^.

Simultaneously alongside those ancient carnivores, many African and Asian newcomers are present, whose presence is very useful for the biochronological analysis. The primitive dhole *Cuon alpinus priscus*, found in layer J2, appeared for the first time in Europe during MIS 15^35^. A broad analysis of the Eurasian material of the Pleistocene lion showed that the morphology of *Panthera spelaea fossilis* from Tunel Wielki Cave corresponds to those dated to MIS 20–12 (Fig. 8)^72^. Its remains were found in all the layers of lithological Series II as well as layer G and in colluvial layers E and L1 (Fig. 3).

A faunal turnover can be observed in the cold period of MIS 12. Tundra species expanded toward the south and south-western Europe, entering the newly formed tundra-steppe ecosystem. At the same time, Asian steppe species dispersed towards the north and north-west. The changes led to the formation of the earliest pan-Eurasian Mammoth Fauna ca 460 ka^73^, which dominate the late Middle Pleistocene and early Late Pleistocene large mammal assemblages. Among such species in Tunel Wielki Cave we have *Gulo gulo* in layer M2, with similarly dated records from Mosbach 3^74,75^ and *Ursus arctos priscus*, found in layers K1, M2 and O, which since MIS 12 was a widespread and permanent member of tundra-steppe palaeocommunities^76^. MIS 12 is also the time of the first appearance of *Martes martes*, also correlated with the boreal taiga habitats^77,78^. It was found in layer J1. Finally, in layer J2 remains of *Mustela nivalis* appeared, which evolved from *Mustela praenivalis* Kormos, 1934 between MIS 15–13^77,78^. All these data allow us to generally narrow the timespan of the carnivore fauna from lithological Series II of Tunel Wielki Cave to MIS 14-12 (Fig. 8)^49^. However, the taxa documented in wood charcoal assemblages from this series might suggest the occupation during a warmer period (MIS 13).

On the contrary, the U-series dating indicates a later age for the sequences. Considering the taxonomical identification of the tooth as belonging to *Ursus deningeri,* together with a high degree of bone mineralization, we should question the obtained U/Th dating result. We assume that, in this case, U-series dating does not reflect an exact age. The reason behind this might be an open uranium system, i.e., the constant availability of uranium ions in the ambient sediments, which resulted in the continuous uptake by bone of U from the environment. In such cases, the U-series dates have only a *terminus ante quem* significance, as was already identified in the nearby Koziarnia Cave^54^.

The timing of the hominin migrations to Central Europe is still under debate^3,79,80^. The earliest confirmed traces, dated to MIS 15-13, are known from Korolevo, Level VI and Miesenheim^14,81–83^). However, isolated stone artefacts were found in Level VII at Korolevo, dated to 0.95 Ma^14,82^. The possibility of an even earlier hominin migration to Central Europe have also arisen, along with new data published from Kozarnika Cave in Bulgaria^1^.

More stone assemblages can be dated to MIS 12-11, including Kärlich-Seeufer, Vértesszölös 2, Rusko 42 or MIS 11-9 i.e. Bilzingsleben, Mauern, Schöningen 13, Trzebnica 2^21,23,26–28,84,85^ Such a chronology has also been recently proposed for two Bochemian open-air sites (Račinĕves and Karlštejn-Altán) by Svoboda & Horáček^86^ and Medzhibozh 1 in Ukraine^87,88^.

Middle Pleistocene stone assemblages in Central Europe are based on flake technology with almost no use of bifacial technology, which prevails in the so-called Acheulean assemblages in western Europe^6,14,19,79,89,90^. Apart from Central Europe, similar flake inventories are known from the Italian peninsula^4,91–95^, Greece^96^ and the Levant, among others (for further discussion see^97^). Some researchers emphasize the small size of the debitage and the accompanying toolkit as a distinct feature of the Central European flake assemblages, and tend to call them microlithic industries or micro-tool^79,84,96–100^ industries. In the case of the Tunel Wielki Cave assemblage, the average size of the debitage is relatively bigger than the other flake industries (Supplement Table S13). We can assume that the abundance of raw material might be the reason for the relatively large size of the debitage compared to other Central European sites, but technological differences might also be the reason.

Detailed technological analyses of the flake assemblages from Central Europe show some chronological discrepancies. As analysed by Rocca^79^, the pre-Holsteinian assemblages such as Korolevo level VI or Kärlich-Seeufer are focused on backed flake production with scarce traces of further edge modifications. In contrast, the latter Holsteinian assemblages, such as Vértesszölös and Bilzingsleben, tend to focus on small thin triangular debitage, which can be modified further. The prevalence of backed flakes in the Tunel Wielki Cave assemblage shows similarities to the first group of sites.

The other interesting issue is the suggested dominance of the bipolar-on-anvil knapping mode in Tunel Wielki Cave. This knapping mode is the simplest one, recognized already in the Oldowan assemblages, and is traditionally ascribed to Mode 1 technology^101–109^. In European sites, the bipolar-on-anvil technique prevails at the earliest Lower Palaeolithic sites, e.g. Fuente Nueva, Barranco León^110^ and Vallparadis^104,111^. It also appears in later Acheulean and flake assemblages but rarely as a prevailing knapping mode^4,112–116^. The only site with the dominant use of the bipolar-on-anvil mode dated later than MIS 16 is the Isernia La Pineta site^92,117–119^.

In most cases, this knapping mode was used for knapping raw materials of poor quality, such as quartz, quartzite or raw material nodules with internal fractures^4,104,112,115,120,121^. In the case of Tunel Wielki Cave, it was used for flint nodules of relatively good knapping quality. Additionally, the abundance of raw material in the direct vicinity of the cave indicated that the knapping mode was not due to raw material shortages and the small size of the cores, as suggested in the case of Bizat Ruhama^122,123^. Interestingly, a similar relationship between the knapping mode and use of local flint as the predominant raw material was identified at Isernia La Pineta^92^.

Remarkably, the traces of Middle Pleistocene human occupation at cave sites are relatively scarce and concentrated in south-western Europe^2,3,114,124,125^ (Fig. 1). In Central Europe, the only evidence of cave use comes from the lowermost layers in Biśnik Cave, located in the northern part of Kraków Upland, ca. 50 km to the north from Tunel Wielki Cave^32,126^. Still, we should stress the lack of bifacial technology and the prevalence of big robust flakes in the assemblage^33^.

Therefore, one can ask why the cave occupation traces are so scarce in Central Europe. The geological processes and erosion of Middle Pleistocene sediments from cave sites might be possible explanations (see^36^ for further discussion). At the same time, human occupation in Central European caves might have been restricted due to humidity and temperature conditions in the caves. As long as the cave temperature resembles the mean annual temperature in the region^127^, we may assume that Central European caves were less likely to have been preferred settlement places than the southern European ones. High humidity in the caves might have been an additional disadvantage for cave occupation.

Cave occupation would require extensive use of fire, as discussed elsewhere^128^. A review of all available traces of intentional fire use at Lower Palaeolithic sites by Roebroeks and Vill^129^ indicates how scarce they are. It is considered that the control of fire became an important part of the technological repertoire of hominins that occupied Europe in the period around 300–400 ka^129^. New research from the Portuguese site of Gruta da Aroeira also testifies to the early use of fire around 400 ka, correlated with MIS 11^125^. The use of fire was also confirmed at other Central European sites with flake assemblages, i.e. Bilzingsleben, Schöningen 13 and Vértesszölös 2, due to the presence of artefacts with traces of ancient heating episodes or burnt bones^129^. The recent analyses conducted at Schöningen 13 ruled out human impact on fire use at the site, showing that macroscopic evidence might not be enough to rule out natural fire episodes^130^. New findings from the site of Cueva Negra del Estrecho suggest that hominins from Europe had the ability to use fire even earlier, in the late Early Pleistocene, corresponding to 800 ka^131,132^.

Interestingly, a single flake from Tunel Wielki Cave shows traces of heating. It is unevenly covered by a mesh of cracks. In Tunel Wielki Cave, no combustion structure has been found. Based on micromorphological analysis of the sediments of layer F there was no trace of fire use. However, the above-mentioned previously heated flake found in layer F may indicate the presence of fire in the cave. The small assemblage of wood charcoal remains could have also been related to the fire activities of the cave inhabitants. Further studies are require in order to confirm the Middle Pleistocene use of fire in Tunel Wielki Cave.

In conclusion, the obtained results indicate that human groups penetrated the Central European Uplands, even north of the Carpathians, during MIS 14-12. It is a matter for further studies to analyse to what extent the climatic and paleoenvironmental conditions in the region enforced the observed technological changes.

## Materials and methods

The 1967–68 fieldwork covered 20 m^2^ of the northern chamber. The eastern and western sides of the chamber were excavated separately as two trenches. As a consequence, a longitudinal and transversal cross-section could be documented. Sediments were excavated in artificial 10 cm thick layers, and artefacts were documented according to were documented using a 1 m2 grid. A plan of the whole trench was prepared after every 10 cm. Animal bones from the same layer/depth/square metre were collected together. Due to their scarcity, stone artefacts were collected separately and indicated on the plan of the trench.

The fieldwork in 2018 covered 2 m^2^ of previously unexcavated surface and 4 m^2^ of the old trenches. The sediments were excavated with 10 cm artificial layers. Artefacts and bones were documented in situ in 3 dimensions using a total station. Big specimens were measured with the use of 2 points. The whole sediment was additionally wet-sieved using a 1 mm mesh.

Additionally, the backfill of the old trenches was excavated using archaeological methods and wet sieved using a 3 mm mesh. Each bucket of sediments was catalogued and wet-sieved separately to avoid contamination. In total, 928 soil buckets of 20 l, including 410 buckets of in situ sediments, were collected and wet-sieved.

### Sedimentology

The sedimentology and lithology of the Tunel Wielki Cave sediments have been presented in detail elsewhere^41^. As T. Madeyska's description was published only in Polish and in a monograph with limited availability, a summary of this study is also provided (see: Supplement Table S3 and Fig. 3).

Thanks to the 2018 excavation campaign, the sediments were available for additional sampling. The micromorphological approach was applied to identify potential micro-scale depositional and postdepositional features preserved in the sediments. Several blocks of undisturbed sediment were collected from the East and North section (Fig. 3), covering layers E, E–F–G–H, and K1.

Preparation of thin sections followed the protocol presented by Krajcarz et al.^42^ and Morley et al.^133^. Micromorphological features were analysed under a polarising microscope according to the well-established guidelines^134^.

### Geochronology

#### OSL

A single sediment sample collected from a loess stratum – layer C – in 2018 was dated using the OSL method. A detailed description of the methods is presented in Supplement Methods S5.

#### U-series

A single animal tooth collected in 1968 from Layer M (Supplement Table S6) was dated using the U-series method. The chemical procedure was conducted in the U-series Laboratory of the Institute of Geological Sciences, Polish Academy of Sciences in Warsaw. The method included the thermal decomposition of organic matter and adding the ^233^U-^236^U-^229^Th spike to the samples, which was then dissolved in nitric acid. Uranium and thorium were separated from the hydroxyapatite matrix using the chromatographic method with TRU-resin^135^. The isotopic composition of U and Th was measured in the Institute of Geology, Czech Academy of Sciences in Prague, with the use of a double-focusing sector-field ICP mass analyser (Element 2, Thermo Finngan MAT) at low mass resolution (m/Δm ≥ 300). The measurements were corrected further to include background and chemical blanks in the calculations. The age errors were calculated using error propagation rules considering all uncertainties except the decay constant.

### Lithic assemblage

Lithic analyses covered stone artefacts found during the fieldwork conducted in 1967–1968 and 2018. According to Chmielewski's field inventory of finds, during the 1967–1968 campaign, 43 stone artefacts, mostly flakes and cores, were found in the Pleistocene loams, which have never been analysed or published before.

Chmielewski found the majority of stone artefacts in so-called layer 10 (n = 26), and this stratum was initially identified as an artefact-bearing layer^41^. However, 13 artefacts were also discovered in loamy strata located below layer 10. Additionally, in the overlying stratum 11, four artefacts of a similar state of preservation were found. Additionally, a single flake was found in the lowermost loess sediments in the southern chamber; this flake is of a similar state of preservation for the surfaces and edges. All artefacts are present in the collection and are available for further studies.

Two lithic artefacts were found in the Pleistocene layers excavated during fieldwork in 2018; one in layer F, which refers to Madeyska's layer 10^41^, and a second in layer E = Madeyska's layer 11^41^. During the wet sieving of the sediments, a further 116 small lithics, mostly chips and chunks, were found. Twelve artefacts were found within layer F, and 48 from loamy layers located under layer F. The distribution of artefacts within the layers is presented in Supplement Table S12. Exploration and wet sieving of the backfill of the old trenches yielded an additional nine lithic artefacts with a similar state of preservation.

Even though layer F was originally determined to be an artefact-bearing stratum, multiple small pieces were found, especially during wet sieving and in the underlying strata. The state of preservation of these artefacts differs substantially from the ones found within the Late Pleistocene and Holocene layers. For this reason, it was decided to analyse all the artefacts found within the loamy strata to check their morpho-technological coherence. In total, the analysed lithic assemblage consists of 171 pieces.

In order to differentiate between artefacts and geofacts, a list of basic morpho-technological features was used. For details, check Lubinsky et al^136^.

The artefacts which fulfilled all the basic features were determined as flakes/chips. Artefacts that were either too small to identify their dorsal and ventral side or did not have them were categorized as chunks. As long as flint nodules do not occur naturally in the caves, raw material nodules with single scars without an identified striking platform and working surface were also identified as chunks. The division between flakes and chips was based on dimensions but also technological traits. Chips are artefacts smaller than 10 mm, whose technological function cannot be determined.

Flakes were additionally described according to the 33 detailed morphological and technological features (Supplement Table S11). The attributes could be divided into four general groups:

- general artefact morphology (the size, shape, state of preservation/fragmentation, symmetry, cross-section, profile, the character of the distal part),
- the condition of the dorsal face (the direction of the scars, cortex, interscar ridges, erasing chips, retouch),
- the condition of the ventral face (the bulbs, bulbar scars),
- the condition of the butt (the size, shape, profile, preparation).

In order to differentiate between the postdepositional and intentional edge retouches, several features were taken into consideration following the general scheme accepted in the literature^55,137,138^. Only a continuous series of edge removals of similar morphology, angle and range was identified as an intentional retouch. All the edge removals detached alternately, non-continuously, or that changed the edge parameters randomly were identified as postdepositional edge damage.

Ten cores were analysed using a scar pattern analysis following the recognized procedures^139,140^. The methods aimed to identify the knapping techniques used and to reconstruct the knapping scheme.

### Palaeontological remains

Large mammal remains, mainly carnivores, were studied in detail elsewhere^35,48,49,72^. For the recent papers, all the identified remains were attributed and divided by layers to check the working hypothesis of the Middle Pleistocene chronology of the layers containing archaeological artefacts. The distribution of the bones within the Pleistocene layers is presented in Supplement Table S16.

Bird remains are very scarce; the methods used for their study and the obtained results are presented in Supplement Data S14. Mollusc remains were extracted from the sediments collected in 2018 (layers G/H, I, J2, M2) and washed through a 1 mm mesh. They were taxonomically determined under a binocular microscope at magnifications up to 65 × , according to Kerney et al.^141^ and Wiktor^142^. The shell material is extremely scarce, comprising only single shells (or shell fragments) assigned to 4 taxa (Supplement Data S15). Some have signs of chemical and/or mechanical dissolution, whereas others are well preserved. Mollusc remains are stored at the Faculty of Geology, University of Warsaw.

### Anthracology: taxonomic identification

The charcoal assemblage from Tunel Wielki Cave came from 17 wet-sieved samples taken from various layers of Pleistocene origin. The wood anatomy of the charcoal fragments was studied with a reflected light microscope with magnifications of 100, 200 and 500 to observe three anatomical sections of the wood in freshly broken charcoal fragments. Taxonomical identifications were made by comparing the specimens with the modern wood collections of the Palaeobotany and Palaeoenvironment Group of the W. Szafer Institute of Botany PAS and atlases of wood anatomy^143,144^. The species of the majority of trees and shrubs cannot be differentiated within their genera. Therefore, the identification of all taxa is limited to the genus level. The wood anatomy of *Picea* and *Larix* is very similar. Thus, both genera are included in one taxon. The same is true for the other two genera, *Salix* and *Populus*, which cannot be differentiated in some cases. Coniferous wood was indicated when cross-field details could not be observed due to a bad preservation state.

## Supplementary Information

Supplementary Information.

## Data Availability

The authors confirm that the data supporting the findings of this study are available within the article and its supplementary materials.
