# Supplementary material for: Frontiers of the Lower Palaeolithic expansion in Europe: Tunel Wielki Cave (Poland)
Source: Sci Rep. 2022 Sep 29;12:16355. doi: 10.1038/s41598-022-20582-0 (PMC9523034; doi:10.1038/s41598-022-20582-0)
Supplement: Supplementary file 1 — Supplementary Information. [file 41598_2022_20582_MOESM1_ESM.pdf]

Supplement Table S1 *List of MIS 13-11 sites in Europe.*

No	Site	Country	Age	References
1	Tunel Wielki Cave	Poland	MIS 14-12	this paper
2	Trzebnica 2	Poland	MIS 13-11	[1]
3	Rusko 33, 36 & 42	Poland	MIS 11	[2]
4	Medzhibozh 1	Ukraine	MIS 11	[3,4]
5	Korolevo VI	Ukraine	MIS 15/13	[5,6]
6	Vértesszölös 2	Hungary	MIS 12/11	[7]
7	Bilzingsleben	Germany	MIS 11/9	[8–11]
8	Racineves	Czechia	MIS 11	[12]
9	Karlštejn-Altán	Czechia	MIS 11/9	[13]
10	Misenheim	Germany	MIS 13	[14,15]
11	Karlich-Seeufer	Germany	MIS 11	[16,17]
12	Waverly Wood	Great Britain	MIS 13	[18]
13	Happisburgh Site 1	Great Britain	MIS 13	[19]
14	Warren hill	Great Britain	MIS 13/12	[20]
15	High Lodge	Great Britain	MIS 13	[21]
16	Beeches Pit	Great Britain	MIS 11	[22,23]
17	Hoxne	Great Britain	MIS 11	[24,25]
18	Elveden	Great Britain	MIS 11	[26]
19	Barnham	Great Britain	MIS 11	[27,2826]
20	Clacton-on-Sea	Great Britain	MIS 11	[29]
21	Swanscombe	Great Britain	MIS 11	[30]
22	Boxgrove	Great Britain	MIS 13	[31,32]
23	Cagny-la-Garenne	France	MIS 12	[33]
24	Ferme de l'Epinette	France	MIS 11/10	[34]
25	"Rue de Cagny"	France	MIS 11/10	[35]
26	Saint-Pierre-les-Elbeuf	France	MIS 11	[36]

27	Menez Dregan	France	MIS 11	[37,38 37]
28	La Celle	France	MIS 11	[39]
29	St. Colomban	France	MIS 11	[40]
30	La Grande vallé	France	MIS 13-11	[41]
31	Terra Amata	France	MIS 11	[42]
32	Arago	France	MIS 14-10	[43]
33	Atapuerca Galeria	Spain	MIS 12-9	[44,45]
34	Aridos	Spain	MIS 11/9	[46]
35	Ambrona	Spain	MIS 11	[47,48]
36	Gruta da Aroeira	Portugal	MIS 11	[49,50]
37	Visogliano	Italy	MIS 13/11	[51]
38	Ficoncella	Italy	MIS 13	[52]
39	Fontana Ranuccio	Italy	MIS 12	[53,54]
40	Castel di Guido	Italy	MIS 11/9	[55,56]
41	Valle Giumentina	Italy	MIS 15-12	[57]
42	Isernia-la-Pineta	Italy	MIS 15	[58–60]
43	Guado San Nicola	Italy	MIS 11/10	[61]
44	Marathousa	Greece	MIS 12/11	[62,63]
45	Dealul Guran	Romania	MIS 11	[64,65]

References

1. Burdukiewicz, J. M., Snieszko, Z. & Winnicki, J. A lower Palaeolithic settlement at Trzebnica (SW Poland). *Ethnogr. Zeitschrift* **35**, 27–40 (1994).

2. Burdukiewicz, J. M. Lower Paleolithic sites with small artefacts in Poland. in *Lower Paleolithic Small Tolls in Europe and the Levant, 1115* (eds. Burdukiewicz, J. M. & Ronen, A.) 65–92 (Archaeopress, British Archaeological Report International Series, 2003).

3. Stepanchuk, V. N. & Moigne, A.-M. MIS 11-locality of Medzhibozh, Ukraine: Archaeological and paleozoological evidence. *Quat. Int.* **409**, 241–254 (2016).

4. Stefaniak, K. *et al.* Middle Pleistocene fauna and palaeoenvironment in the south of Eastern Europe: a case study of the Medzhybizh 1 locality (MIS 11, Ukraine). *Quat. Int.* doi:10.1016/j.quaint.2021.07.013.

5. Haesaerts, P. & Koulakovska, L. La séquence pédosédimentaire de Korolevo (Ukraine transcarpatique): contexte chronostratigraphique et chronologique. in *The European Middle Palaeolithic* (ed. Kulakovska, L. V.) 21–37 (Institut d’Archeologie de l’Academie des Sciences

d'Ukraine, 2006).

6. Koulakovska, L., Usik, V., Haesaerts, P. Early Paleolithic of Korolevo site (Transcarpathia, Ukraine). *Quat. Int.* **223**–**224**, 116–130 (2010).

7. Kretzoi, M. & Dobosi, V. *Vértesszőlős – Man, Site, Culture*. (Akadémiai Kiadó, 1990).

8. Mania, D. *Auf den Spuren des Urmenschen. Die Funde aus der Steinrinne von Bilzingsleben*. (Deutscher Verlag der Wissenschaften, 1990).

9. Beck, M., Gaupp, R., Kamradt, I., Liebermann, C. & Pasda, C. Bilzingsleben site formation processes e geoarchaeological investigations of a middle pleistocene deposit: Preliminary results of the 2003-2005 excavations. *Archaol. Korrespondenzblatt* **37**, 1–18 (2007).

10. Müller, W. & Pasda, C. Site formation and faunal remains of the middle pleistocene site Bilzingsleben. *Quartar* **58**, 25–49 (2011).

11. Bock, C., Neubeck, V. & Pasda, C. Non-flint from the Middle Pleistocene site of Bilzingsleben (excavation from 1971 to 2002). Quartär **64,** 7–25 (2017).

12. Tyráček, J., Westaway, R. & Bridgland, D. River terraces of the Vltava and Labe (Elbe) system, Czech Republic, and their implications for the uplift history of the Bohemian Massif. *Proc. Geol. Assoc.* **115**, 101–124 (2004).

13. Svoboda, J. & Horáček, I. Between Bilzinbsleben and Vértésszölös: small-sized industries in the middle of Europe (Czech Republic). *Anthropol.* **57**, 363–371 (2019).

14. Turner, E. Miesenheim I: a lower palaeolithic site in the middle Rhineland (Neuwied Basin) FRG. *Ethnogr. Zeitschrift* **30**, 521–531 (1989).

15. Turner, E., Bittmann, F., Beonigk, W. & Frechen, M. *Miesenheim I: Excavation at a Lower Palaeolithic Site in the Central Rhineland of Germany*. (Verlag des Römisch-Germanischen Zentralmuseums, 2000).

16. Gaudzinski, S. *Karlich-Seeufer: Untersuchungen zu einer Altpalaolithischen Fundstelle in neuwieder Becken (Rheinland-Plalz)*. *Jahrbuch des Romische-Germanischen Zentralmuseums* (Verlag des Römisch-Germanischen Zentralmuseums, 1996).

17. Gaudzinski, S., Bittman, F., Boenigk, W., Frechen, M. & Van Kolfschoten, T. Palaeoecology and archaeology of the karlich-aeeufer open-air site (middle pleistocene) in the Central Rhineland, Germany. *Quat. Res.* **46**, 319–334 (1996).

18. Shotton, F. W. *et al.* The middle pleistocene deposits of Waverley Wood Pit, Warwickshire, England. *J. Quat. Sci.* **8**, 293–325 (1993).

19. Parfitt, S. A. *et al.* Early pleistocene human occupation at the edge of the boreal zone in northwest Europe. *Nature* **466**, 229–233 (2010).

20. Wymer, J. J., Lewis, S. G. & Bridgland, D. R. Warren Hill, Mildenhall, Suffolk (TL 744743). in *Central East Anglia and the Fen Basin. Field Guide* (eds. Lewis, S. G., Whiteman, C. A. & Bridgland, D. R.) 50–58 (Quaternary Research Association, 1991).

21. Bowen, D.Q. *High Lodge: Excavations by G. de G. Sieveking 1962-68 and J. Cook 1988*. (British Museum Press, 1992).

22. Preece, R. C., Gowlett, J. A. J., Parfitt, S. A., Bridgland, D. R. & Lewis, S. G. Humans in the Hoxnian: habitat, context and fire use at Beeches Pit, West Stow, Suffolk, UK. *J. Quat. Sci.* **21**, 485–496 (2006).

23. Gowlett, J. A. J., Hallos, J., Hounsell, S., Brant, V. & Debenham, N. C. Beeches Pit: archaeology, assemblage dynamics and early fire history of a Middle Pleistocene site in East Anglia, UK. *J. Eurasian Prehistory* **3**, 3–38 (2005).

24. Ashton, N. M., Lewis, S. G., Parfitt, S. A., Penkman, K. E. H. & Coope, G. R. New evidence for complex climate change in MIS 11 from Hoxne, UK. *Quat. Sci. Rev.* **27**, 652–668 (2008).

25. Singer, R., Gladfelter, B. G. & Wymer, J. J. *The Lower Paleolithic Site at Hoxne, England*. (University of Chicago Press, 1993).

26. Ashton, N. *et al.* Excavations at the Lower Palaeolithic site at Elveden, Suffolk, UK. *Proc. Prehist. Soc.* **71**, 1–61 (2005).

27. Ashton, N., Lewis, S.G. & Parfitt, S. (eds.) *Excavations at Barnham, 1989-94. British Museum Occasional Paper 125*. (British Museum Occasional Paper 125, 1998).

28. Ashton, N., Mcnabb, J., Irving, B., Lewis, S. & Parfitt, S. Contemporaneity of Clactonian and Acheulian flint industries at Barnham, Suffolk. *Antiquity* **68**, 585–589 (1994).

29. Bridgland, D. R. *et al.* Middle Pleistocene interglacial Thames-Medway deposits at Clacton-on-Sea, England: Reconsideration of the biostratigraphical and environmental context of the type Clactonian Palaeolithic industry. *Quat. Sci. Rev.* **18**, 109–146 (1999).

30. White, T. S., Preece, R. C. & Whittaker, J. E. Molluscan and ostracod successions from Dierden's Pit, Swanscombe: insights into the fluvial history, sea-level record and human occupation of the Hoxnian Thames. *Quat. Sci. Rev.* **70**, 73–90 (2013).

31. Pope, M. & Roberts, M. Observations on the relationship between palaeolithic individuals and artefact scatters at the middle pleistocene site of Boxgrove, UK. in *Hominid Individual in Context: Archaeological Investigations of Lower and Middle Palaeolithic Landscapes, Locales and Artefacts* 81–97 (2005). doi:10.4324/9780203007693.

32. Roberts, M. B. & Parfitt, S. A. *Boxgrove: a Middle Pleistocene hominid site at Eartham Quarry, Boxgrove, West Sussex*. (English Heritage, 1999).

33. Antoine, P. & Tuffreau, A. Contexte stratigraphique, climatique et paléotopographique des occupations acheuléennes de la moyenne terrasse de la Somme. *Bull. la Société préhistorique française* **90**, 243–250 (1993).

34. Tuffreau, A., Lamotte, A. & Marcy, J. L. Land-use and site function in acheulean complexes of the somme valley. *World Archaeol.* **29**, 225–241 (1997).

35. Davis, R. & Ashton, N. Landscapes, environments and societies: The development of culture in Lower Palaeolithic Europe. *J. Anthropol. Archaeol.* **56**, 101107 (2019).

36. Cliquet, D. *et al.* Loessic sequence of saint-pierre-les-elbeuf (Normandy, France): New archaeological, geochronogical and palaeontological data. *Quaternaire* **20**, 321–343 (2009).

37. Ravon, A. L., Monnier, J. L. & Laforge, M. Menez-Dregan I, layer 4: A transitional layer between the Lower and Middle Palaeolithic in Brittany. *Quat. Int.* **409**, 92–103 (2016).

38. Ravon, A.-L. & Monnier, J.-L. La transition Paleolithique inferieur-moyen dans l'Ouest armoricain: l'exemple de la couche 4 du site de Menez-Dregan I (Plouhinec, Finistere). *Bull. la Soc. Prehist. française* **110**, 5–21 (2013).

39. Limondin-Lozouet, N. *et al.* Oldest evidence of Acheulean occupation in the Upper Seine valley (France) from an MIS 11 tufa at La Celle. *Quat. Int.* **223**–**224**, 299–311 (2010).

40. Cloirec, R. Le & Monnier, J.-L. Le gisement paléolithique inférieur de La Pointe de Saint-

Colomban, Carnac (Morbihan). *Gall. Préhistoire* **28**, 7–36 (1985).

41. Hérisson, D., Airvaux, J., Lenoble, A. & Richter, D. The Acheulean site of "La Grande Vallée" at Colombiers (Vienne, France): stratigraphy, formation processes, preliminary dating and lithic industries. *Paleo* **23**, (2012).

42. de Lumley, H., Khatib, S., Echassoux, A. & Todisco, D. Les lignes de rivage quaternaires en relation avec les sites paléolithiques des Alpes-Maritimes et de la Ligurie occidentale. *ArchéoSciences, Rev. d'archéométrie* **25**, 125–134 (2001).

43. Barsky, D. & Lumley, H. de. Early European Mode 2 and the stone industry from the Caune de l'Arago's archeostratigraphical levels 'P.' *Quat. Int.* **223**–**224**, 71–86 (2010).

44. Falguères, C. *et al.* Combined esr/u-series chronology of acheulian hominid-bearing layers at trinchera galería site, atapuerca, spain. *J. Hum. Evol.* **65**, 168–184 (2013).

45. Ollé, A. *et al.* The Early and Middle Pleistocene technological record from Sierra de Atapuerca (Burgos, Spain). *Quat. Int.* **295**, 138–167 (2013).

46. *Ocupaciones achelenses en el valle del Jarama (Arganda, Madrid)*. (Publicaciones de la Diputación Provincial, 1980).

47. Santonja, M. *et al.* Ambrona and Torralba archaeological and paleontological sites. The Current Archaeological Record. in *Pleistocene and Holocene Hunter-Gatherers in Iberia and the Gibraltar Strait* (ed. Sala, R.) 517–527 (Universidad de Burgos, Fundación Atapuerca, 2014).

48. Santonja, M., Rubio-Jara, S., Panera, J., Sánchez-Romero, L. Tarriño, A. & Pérez-González, A. Ambrona revisited: The Acheulean lithic industry in the Lower Stratigraphic Complex. *Quat. Int.* **480**, 95–117 (2018).

49. Daura, J. *et al.* New Middle Pleistocene hominin cranium from Gruta da Aroeira (Portugal). *Proc. Natl. Acad. Sci. U. S. A.* **114**, 3397–3402 (2017).

50. Sanz, M. *et al.* Early evidence of fire in south-western Europe: the Acheulean site of Gruta da Aroeira (Torres Novas, Portugal). *Sci. Rep.* **10**, 12053 (2020).

51. Falguères, C. *et al.* ESR/U-series chronology of the Lower Palaeolithic palaeoanthropological site of Visogliano, Trieste, Italy. *Quat. Geochronol.* **3**, 390–398 (2008).

52. Rocca, R., Abruzzese, C. & Aureli, D. European Acheuleans: Critical perspectives from the East. *Quat. Int.* **411**, 402–411 (2016).

53. Segre, A. G. & Ascenzi, A. Fontana Ranuccio: Italy's earliest Middle Pleistocene hominid site. *Curr. Anthropol.* **25**, 230–233 (1984).

54. Naldini, E. S., Muttoni, G., Parenti, F., Scardia, G. & Segre, A. G. Nouvelles recherches dans le bassin Plio-Pléistocène d'Anagni (Latium méridional, Italie). *Anthropologie.* **113**, 66–77 (2009).

55. Boschian, G. & Saccà, D. In the elephant, everything is good: carcass use and reuse at Castel di Guido (Italy). *Quat. Int.* **361**, 288–296 (2015).

56. Michel, V., Boschian, G. & Valensi, P. Datation ESR de dents d'aurochs du site Paléolithique inférieur de Castel di Guido (Italie). *http://journals.openedition.org/archeosciences* **32**, 51–58 (2008).

57. Villa, V. *et al.* A MIS 15-MIS 12 record of environmental changes and Lower Palaeolithic occupation from Valle Giumentina, central Italy. *Quat. Sci. Rev.* **151**, 160–184 (2016).

58. Vergès, J. M. & Ollé, A. Technical microwear and residues in identifying bipolar knapping on an anvil: Experimental data. *J. Archaeol. Sci.* **38**, 1016–1025 (2011).

59. Gallotti, R. & Peretto, C. The Lower/early Middle Pleistocene small débitage productions in Western Europe: New data from Isernia La Pineta t.3c (Upper Volturno Basin, Italy). *Quat. Int.* **357**, 264–281 (2015).

60. Peretto, C. *et al.* A human deciduous tooth and new 40Ar/39Ar dating results from the Middle Pleistocene archaeological site of Isernia La Pineta, southern Italy. *PLoS One* **10**, e0140091 (2015).

61. Peretto, C. *et al.* The Middle Pleistocene site of Guado San Nicola (Monteroduni, Central Italy) on the Lower/Middle Palaeolithic transition. *Quat. Int.* **PB**, 301–315 (2016).

62. Panagopoulou, E. Tourloukis, V. *et al.* Marathousa 1: a new Middle Pleistocene archaeological site from Greece. *Antiquity* **89**, (2015).

63. Tourloukis, V. & Harvati, K. The Palaeolithic record of Greece: A synthesis of the evidence and a research agenda for the future. *Quat. Int.* **466**, 48–65 (2018).

64. Iovita, R. *et al.* Geoarchaeological prospection in the loess steppe: Preliminary results from the Lower Danube Survey for Paleolithic Sites (LoDanS). *Quat. Int.* **351**, 98–114 (2014).

65. Iovita, R. *et al.* Dealul Guran: evidence for Lower Palaeolithic (MIS 11) occupation of the Lower Danube loess steppe. *Antiquity* **86**, 973–989 (2012).

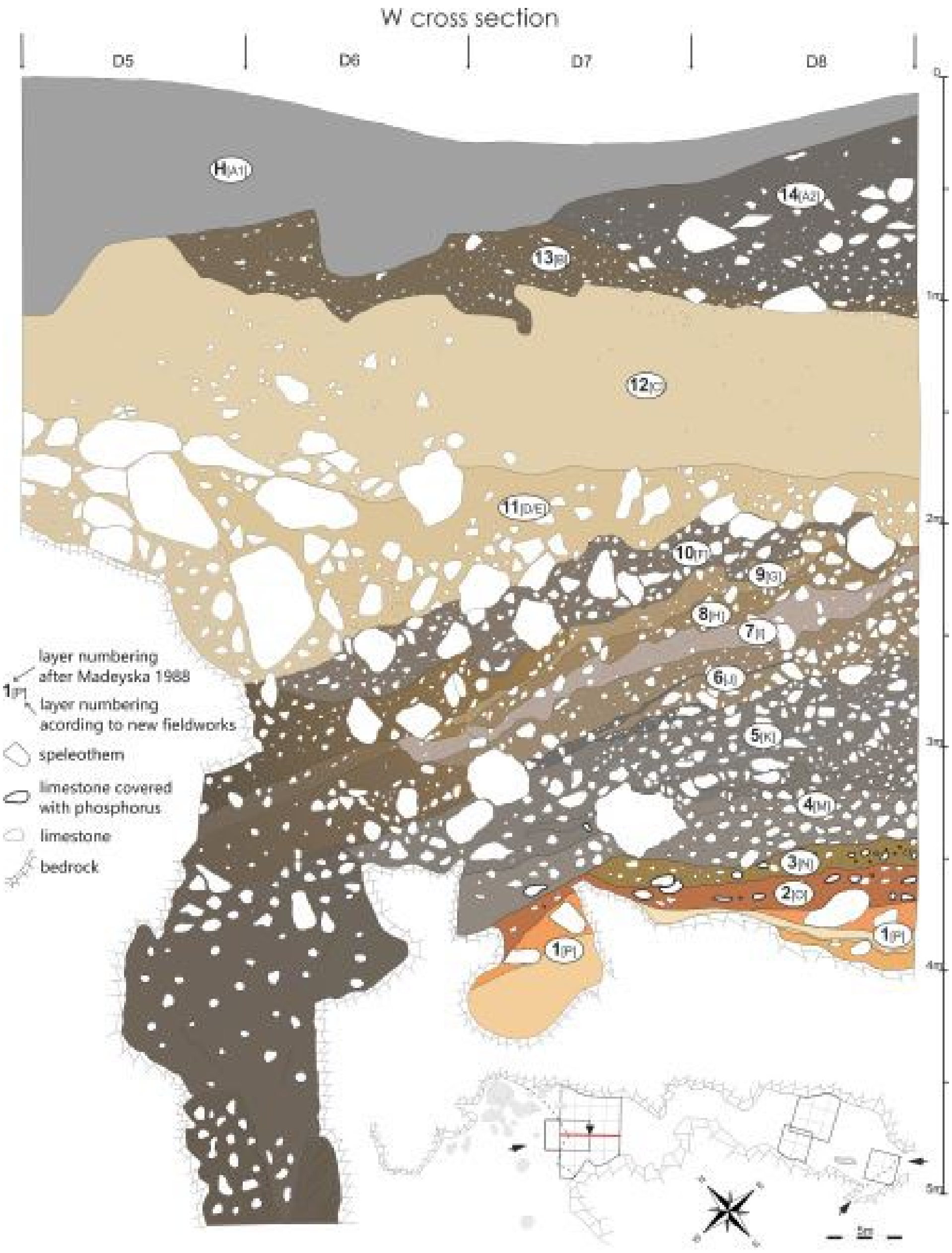

Supplement Fig. S2. *Longitudinal cross-section of the northern chamber of Tunel Wielki Cave obtained during 1967/68 fieldworks. Archaeological artefacts were found mostly in layer F. A vertical chimney in the bottom of the cave can be seen. It caused creeping of the middle part of the section toward the end of the chamber causing postdepositional movments of the layers. (redrawn based on Madeyska*[1] *and fieldwork documentation).*

Supplement Table S3 *Stratigraphic units in Tunel Wielki Cave. Correlation between the 2018 field observation and data from literature and archival documentation is provided, along with the lithological description.*

Layer's numbering, according to:			depth under the surface/ thickness * (cm)	Lithology based on new fieldworks	Depositional environment**
**2018 fieldworks**	**Madeyska (1988)**	**1967-1968 field documentation**			
**h-1**			-/20	the backfill of the old 1968-69 trench; unevenly coloured humus mixed with loams; contains limestone rubble	modern anthropogenic
**h-2**	-	-	-/260	the backfill of the old 1968-69 trench; a mixture of unevenly coloured greyish loams with humus laminae; contains limestone debris	modern anthropogenic
**h-3**			/>50	the backfill of the old 1968-69 trench; a mixture of unevenly coloured reddish loams; contains black manganese clasts and limestone rubble	modern anthropogenic
**A1**	heap	heap	0-20/20	mixed humic sediment, mixed backfield of old trenches	modern anthropogenic
**A2**	14	1, 1s	0-60/50	blackish-grey humus; contains limestone debris	rock falls, phytogenic accumulation, anthropogenic accumulation
**B1**	13	2	50-70/8	blackish humus with an admixture of silt; contains a big amount of limestone debris	rock falls, pedogenesis (illuviation)
**B2**	13	2	50-60/10	milky-brown loamy humus; contains a big amount of limestone debris	
**B3**	13	2	70-80/10	grey-beige, partially reddish sandy loam with a big amount of limestone debris.	washing, rock falls
**C**	12	3	70-160/100	yellowish, partially reddish silt/ silty loam; contains calcite clasts and laminae, especially in its bottom part; does not contain limestone rubble.	eolian (loess)
**D**	12	3	160-180/10	yellowish-grey silt/ silty loam; contains sharp edge limestone debris	eolian, rock falls

**E**	11	4	170-220/10-20	yellowish-grey silty loam; contains a big amount of large limestone debris.	
**F**	10	5	190/10	a dark beige-brown silty loam with limestone clasts and veins; in the bottom part becomes more greyish.	cave floor, zoogenic accumulation, anthropogenic accumulation (based on stone artefacts presence)
**G**	9	6	200/5	yellowish-orange grey compact loam; contains limestone debris	cave floor, zoogenic accumulation
**H**	8	7	220/10	a grey-brown unevenly coloured loam with black discolouration; contains limestone debris	
**I**	7	8	180/10	a dark grey loam with black manganese clasts and limestone debris	
**J1**	6	9	180/10	reddish loam containing limestone debris	
**J2**	6	9	240/10	dark reddish-brown loam; contains a small amount of rounded, chemically eroded limestone debris	cave floor, zoogenic accumulation, chemical weathering
**K1**	5	10	180/20	Dark grey loam of inhomogeneous structure and colourations; contains slightly rounded limestone debris.	
**K2**	5	10	180/30	Dark grey loam of inhomogeneous structure and colourations; contains vacuums of yellowish silt and slightly rounded limestone debris.	
**L1**	5	10	250/15	dark-beige-grey sandy loam of inhomogeneous structure; contains a big amount of small, rounded limestone debris	
**L2**	5	10	250/20	dark-beige-grey sandy loam of inhomogeneous structure; contains a big amount of rounded limestone debris	
**M1**	4	11	280-330/5	dark-grey compact loam of inhomogeneous structure; contains a big amount of limestone debris and different size and limestone powder.	cave floor, chemical weathering
**M2**	4	11	290/15	orange-grey loam of inhomogeneous matrix and multiple discolourations with limestone debris	
**N**	3	12	320-340/20	a dark grey loam with multiple black manganese clasts; contains heavily chemically and mechanically eroded limestone debris	

**O**	2	13	340-400/20	intensively reddish (partially yellowish) loam black-spotted by manganese clasts; contains heavily chemically eroded limestone debris	rock falls, chemical weathering
**P1**	1	14	360-400/5-10	a light grey loam with black manganese clasts; does not contain limestone debris.	chemical weathering
**P2**			350-410/5-15	orange-yellow sandy loam with multiple black manganese clasts and laminae, which change the colouration of the layers into black; does not contain limestone debris.	
**P3**			400-420/10	a dark orange loam with grey discolourations and black manganese clasts; does not contain limestone debris	
**R**	-	13"A"	200-260/60	cracked bedrock filed with yellowish and orange silty loam	physical and chemical weathering

* 2018 fieldworks

**Cave floor environment includes low-rate accumulation by rock fall, followed by physical disintegration of limestone debris and its chemical weathering, with minor input from eolian and biogenic accumulation

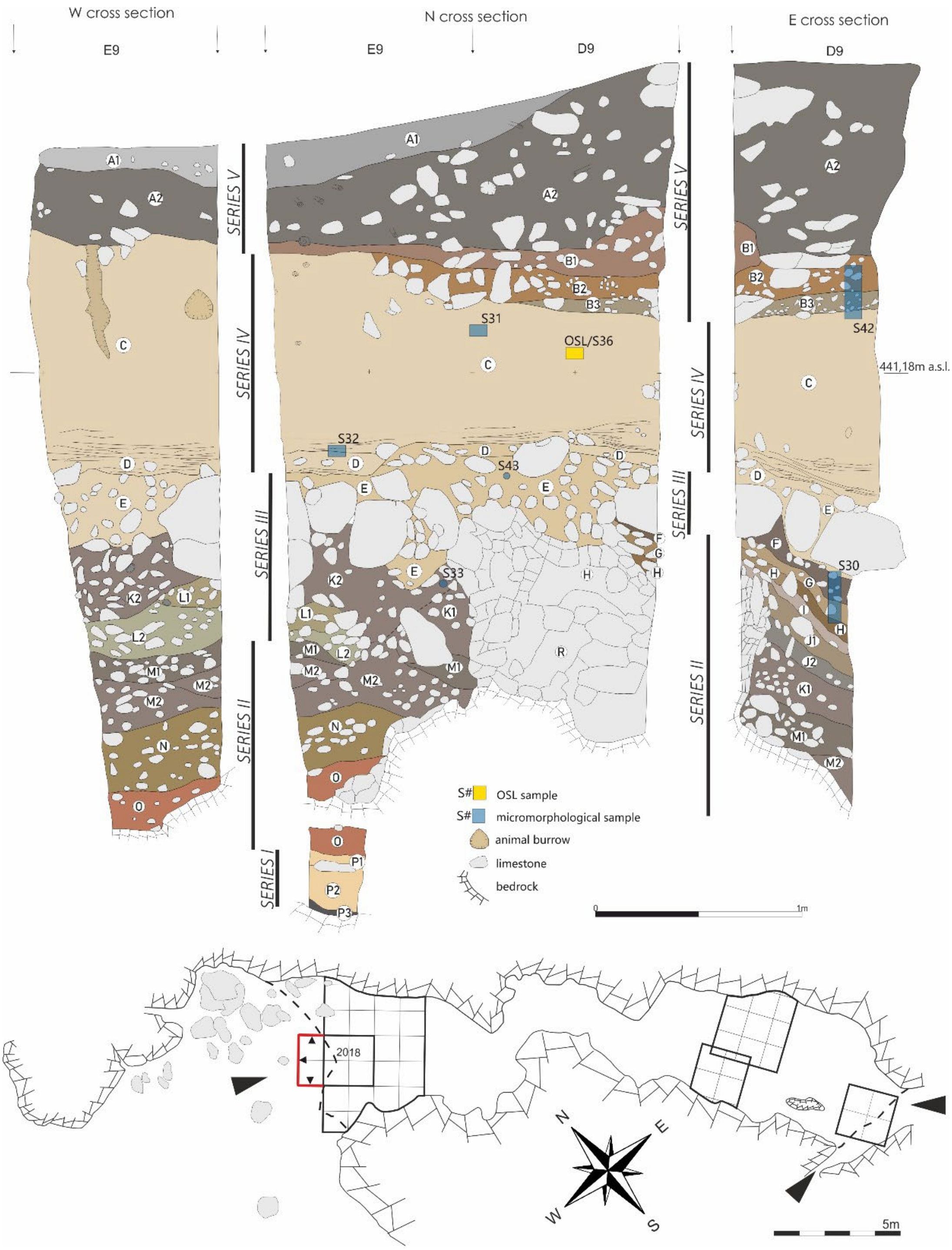

Supplement Fig. S4. *Cross section of 2018 fieldworks with micromorphological and OSL sampling places marked.*

References

1. Madeyska, T. Osady jaskiń i schronisk Doliny Sąspowskiej. in *Jaskinie Doliny Sąspowskiej. Tło przyrodnicze osadnictwa pradziejowego* (ed. Chmielewski, W.) 77–173 (Wydawnictwa Uniwersytetu Warszawskiego, 1988).

Supplement Methods S5

Datings: *OSL dating procedure*

In the laboratory, a sample was dried. High-resolution gamma spectrometry using a HPGe detector manufactured by Canberra was carried out to determine the content of U, Th and K in the samples. Before measurement, the sample was stored for about 3 weeks to ensure equilibrium between gaseous ^{222}Rn and ^{226}Ra in the ^{238}U decay chain. The measurement lasted for at least 24 hours. The activities of the isotopes present in the sediment were determined using IAEA standards RGU, RGTh, RGK after subtraction of the detector background. Dose rates were calculated using the conversion factors of Guerin et al.[1]. For beta dose rate, the cosmic ray dose-rate to the site was determined as described by Prescott and Stephan[2]. We assumed that the average water content was (18±5)%. For further calculations, a mean a-value of 0.08 for fine-grained quartz[3] was used.

For OSL measurements, fine grains of quartz (4–11 µm) were extracted from the sediment samples. The first step was to obtain the fraction below 45 µm using sieves; next, sediments were treated with 20% hydrochloric acid (HCl) and 20% hydrogen peroxide (H_2O_2). Finally, the material was etched by concentrated hydrofluorosilicic acid (34%, H2SiF6) for few days; after that, grains were ready for gravitational separation.

All OSL measurements were made using an automated Risø TL/OSL DA-20 reader. The stimulation light source was a blue (470 ± 30 nm) light-emitting diode (LED) array delivering 50 mW/cm2 at the sample[4]. Detection was through 7.5 mm of Hoya U-340 filter. Equivalent doses were determined using the single-aliquot regenerative-dose (SAR) protocol[5]. The final result was calculated using Central Age Model (CAM) and the equivalent dose distributions[6]. The obtained overdispersion was about 10%.

Details of OSL dating results. Laboratory code of investigated sample, sample name, sample depth (m b. s. = meters below surface), specific activities of natural radionuclides, dose rate, estimated water content, number of measured aliquots, final equivalent dose (CAM model) and calculated age.

Lab, Code	Sample name	Sampling depth (m b,s,)	Th (Bq/kg)	U (Bq/kg)	K (Bq/kg)	Dose rate (Gy/ka)	Water content (%)	Number of measured aliquots	Equivalent dose (Gy)	OSL Age (ka)
GdTL-3199	TW_S38	160 cm	24.03±0.65	22.10±0.36	356±12	1.898±0.63	18±5	13	45.10±0.98	23.69±0.95

Supplement Table S6. *Uranium series dating of Ursus deningeri tooth sample from Tunel Wielki Cave. The reported errors are 2 standard deviations.*

Lab. no.	Sample	U cont. [ppm]	U-234/U-238	Th-230/U-234	Th-230/Th-232	Age [ka]
896	TW 401	1.510±0.007	1.079±0.002	0.358±0.002	62.2±0.3	48.3±0.3

References

1. Guérin, G., Mercier, N. & Adamiec, G. Dose-rate conversion factors: update. *Anc. TL* **29**, 5–8 (2011).
2. Prescott, J. R. & Stephan, L. G. The contribution of cosmic radiation to the environmental dose for thermoluminescence dating. Latitude, altitude and depth dependencies. *TLS* **II–1**, 16–25 (1982).
3. Rees-Jones, J. Optical dating of young sediments using fine-grain quartz. *Anc. TL* **13**, 9–14 (1995).
4. Bøtter-Jensen, L., Bulur, E., Duller, G. A. T. & Murray, A. S. Advances in luminescence instrument systems. *Radiat. Meas.* **32**, 523–528 (2000).
5. Murray, A. S. & Wintle, A. G. Luminescence dating of quartz using an improved singlealiquot regenerative-dose protocol. *Radiat. Meas.* **32**, 57–73 (2000).
6. Berger, G. An alternate form of probability-distribution plots for DE values. *Anc. TL* **28**, 11–22 (2010).

Supplement Data S7

Micromorphological analyses

Layer F was the uppermost loamy layer containing stone artefacts, therefore the analyses focused at answering question about the depositional processes taking place below, above and within layer F. According to Madeyska[1], Layer F is greyish brown loam with rounded limestone clasts and with cultural relics. A presence of large limestone blocks limits the readability of layer's boundaries. T. Madeyska, on the basis of regional scale litho-stratigraphic correlation, linked this layer with an early part of the Last Glaciation (MIS 5d-5a), a relatively warm and humid period, most likely MIS 5a. She linked other loamy layers (G down to N) with the earlier part of MIS 5. Layer F is the last (the uppermost) unit of this loamy series. Its upper boundary is erosional and covered by loess-like sediment of clearly different lithology.

Layer F, together with lower sedimentary units (layers G down to K), shows distinct inclination (around 30° toward E and 30–40° toward S). Analysis of spatial distribution of the mentioned layers reveals, however, that this inclination has limited range. The cross-section achieved by W. Chmielewski and T. Madeyska[1] documents a U-shaped bending of these layers, which more-or-less reproduces the concave morphology of the bedrock. Such a layout may be interpreted as post-depositional plastic deformation likely related to subsidence, which was possibly caused by compaction of underlying sediments, partial dissolution of bedrock and calcareous components of older sediments, and/or presence of empty spaces somewhere below. It is possible that the cave is connected with another cavity called Rockshelter under Tunel Wielki and a Rockshelter above Niedostępna Cave (Fig.2). This is supported by a vertical chimney of ca. 1 m in diameter situated in the bottom of the cave, which was found in the northern chamber[1]. It was excavated down to 1.6 m without reaching the bedrock. It was filled with mixed loamy and silty sediments (Fig.3).

Layer F exhibits typical micromorphological features of sediments deposited in low-energy environment of a cave floor: massive structure, silty clay grain size composition, and presence of numerous bone and tooth fragments (which correlates with other known sites[2–4]). Bone and tooth fragments are chaotically oriented and some of them are in sub-vertical position (Fig.S4: a, b), which likely indicates a re-orientation by some post-depositional agents, such as frost action[5,6] and/or colluvial activity[7,8]. Planar voids are frequent and parallel to each other. Some of them cut through larger grains and aggregates. This suggests that the voids may represent a remnant of ice lenses which are known to produce similar structure[6], so called lenticular microstructure of sediment, and are common in periglacial environments. In opposition to typical periglacial lenticular structure, here the planar voids are inclined. However, their inclination reproduces the general dip of the strata boundaries. This suggest that the voids could possibly have been originally oriented sub-horizontally and were re-oriented together with the entire sediment packet by the subsidence. The inclined orientation of presumably originally horizontal planar voids suggests that the frost action that produced the voids happened before the subsidence. The survival of voids, stratigraphic unit boundaries and other microfeatures indicates that the re-orientation process involved the whole packet of now-inclined layers, without disturbing their inner structure. Similar inclined planar voids occur also within layer G

and weakly developed ones in layer H (Fig.S8: e-h), which indicates that the frost action event affected the entire packet of layers F–G–H. The presence of aggregates or small (c. 1 mm size) diapirs of layer's G just above the layer F/layer G boundary (Fig.S4: c, d) may also be another effect of frost action. Similar plastic deformations at layer boundaries are known from other cave sites [4] and related to frost action.

Original depositional processes cannot be exactly characterized for layer F, as most of original sedimentary features were disturbed by later frost action and subsidence. However, it is clear that the material of layer F is different from the sediments of underlying layers G and H. This dissimilarity is visible in grain size composition (layers G-H much more clayey), b-fabric (birefringence colors much more intense in layers G-H), color (layer F more greyish and layers G–H more brownish in plane polarized light; layer F also paler and less yellowish in cross-polarized light), and abundance of bone and tooth fragments (much more abundant in layer F) (Fig.S8, Table S9). The material of layer F can be therefore regarded as an effect of new depositional event and supply of fresh clastic material, and not any important re-deposition of older sediments. Bone and tooth fragment are abundant in layer F. These are angular fragments, mostly below 0.5 mm in size, with traces of *in situ* cracking (Fig.S8: a, b), which is a phenomenon known from other cave sites and related to frost action[2]. In layer G some bone fragments bear corrosive pits, likely an effect of digesting due to carnivore activity. Such features were not observed in layer F, which may indirectly point toward human-related rather than carnivore-related deposition. On the other hand, no direct micromorphological traces of intense human activity are recorded within the sediment, such as presence of charcoal, char, burnt bones, burnt sediments, or lithic chips (compare with Chagyrskaya Cave[3]).

The upper boundary of layer F is erosional. It is covered by layer E, characterized by several features indicating its colluvial origin: i) unconformity at the bottom; ii) erosional channels locally at the bottom; iii) complex macroscopic texture with clasts of compacted sediments chaotically dispersed within loose matrix; iv) loose microscopic structure with packing voids (Fig.S8: a, b); and v) presence of numerous clay balls, some of them containing bone fragments (Fig.S8: a, b). A hiatus between layers F and E is possible filled by other layers of Series III (layers K2, L1 and L2), present in another part of the site. Layer K2, also analysed micromorphologically, exhibits similar features as the ones observed for layer E (Fig.S8: c, d). Clay balls occurring within layers E and K2 resemble the material of layers F and G. This indicates that layers F and G were amongst the sources of material for colluvial activity, that deposited layer E and other layers of the Series III. This is an additional argument for linking the artefacts found within colluvial sediments of the Series III with artefacts and animal remains preserved in primary position within layer F.

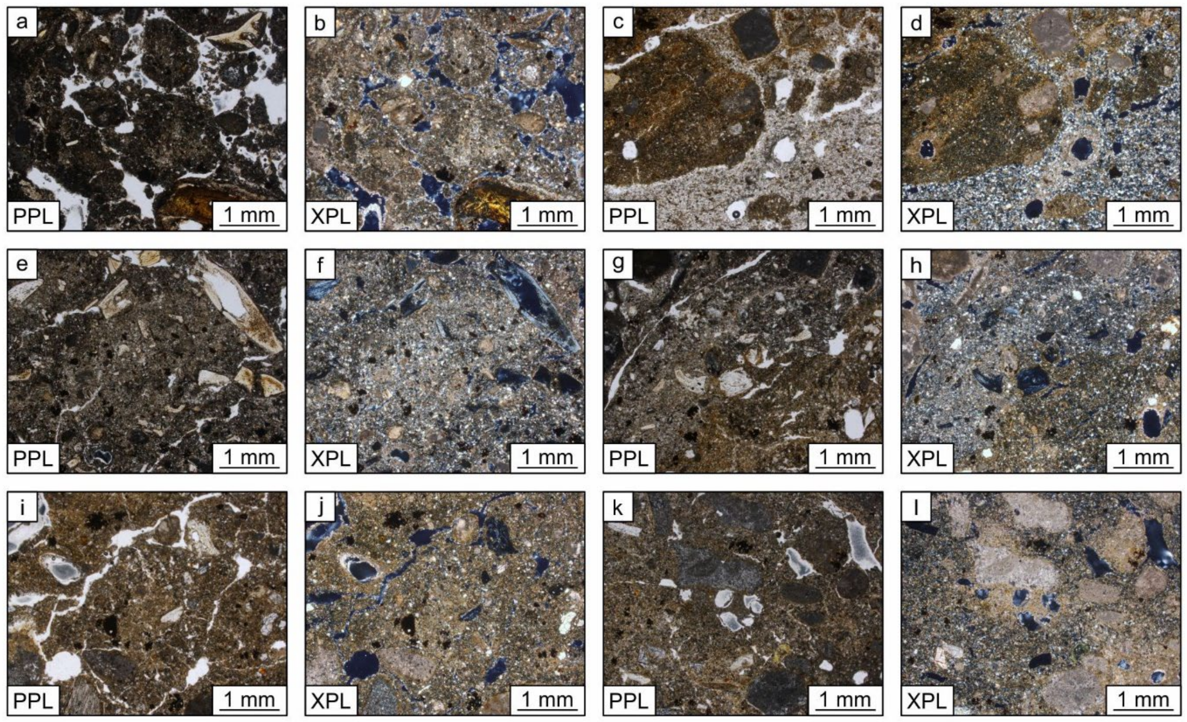

Supplement Fig. S8. Micrographs of sediment from Tunel Wielki Cave: **a, b** – layer E; **c, d** – layer K2; **e, f** – layer F; **g, h** – boundary between layers F and G; **i, j** – layer G; **k, l** – layer H. PPL – plane polarized light; XPL – cross polarized light.

Supplement Table S9. Micromorphological features of Layer F and underlying layers G and H in Tunel Wielki Cave.

Layer	Microstructure	C/f-related distribution	Voids	B-fabric	angular bone/tooth fragments	rounded bone/tooth fragments	corroded bone/tooth fragments	shattered bone/tooth fragments	carbonate impregnations	carbonate hypocoatings	clay balls	sub-vertical orientation of clasts
E	A	Pss, Pds	Pa	S, G	+	+	+	-	-	-	++	-
K2	A, L	Pss, Pds	Ve, Pa	G, S	+	+	-	-	-	-	++	-
F	M, B	Pss	Pl, B	G, S	++	-	+	++	-	+	-	++
G	B, A	Pss	Pl, Ve, B	S, G	++	-	+	+	-	+	+	+
H	M, A	Pss, Pds	Ve, Vu	S	+	-	-	-	++	++	+	+

Microstructure: M – massive, B – blocky, A – aggregative, L – laminate
C/f-related distribution: Pss – porphyric single spaced, Pds – porphyric double spaced
Voids: Pa – packing voids, Pl – planes, B – empty bone interior, Ve – vesicles, Vu – vughs
B-fabric: S – stipple speckled, G – granostriated
Features: - absent, + rare, ++ abundant

References

1. Madeyska, T. Osady jaskiń i schronisk Doliny Sąspowskiej. in *Jaskinie Doliny Sąspowskiej. Tło przyrodnicze osadnictwa pradziejowego* (ed. Chmielewski, W.) 77–173 (Wydawnictwa Uniwersytetu Warszawskiego, 1988).
2. Krajcarz, M. & M.T., K. Post-depositional bone destruction in cave sediments: a micromorphological study of the MIS 5a-d cave bear strata of Biśnik Cave, Poland. *J. Quat. Sci.* **34**, 138–152 (2019).
3. Miller, C. E. *A Tale of Two Swabian Caves: Geoarchaeological Investigations at Hohle Fels an Geissenkloesterle*. (2015).
4. Kolobova, K. A. *et al.* Archaeological evidence for two separate dispersals of Neanderthals into southern Siberia. *Proc. Natl. Acad. Sci. U. S. A.* **117**, 2879–2885 (2020).
5. Cremaschi, M. & Van Vliet-Lanoë, B. Traces of frost activity and ice segregation in Pleistocene loess deposits and till of northern Italy: Deep seasonal freezing or permafrost? *Quat. Int.* **5**, 39–48 (1990).
6. Van Vliet-Lanöe, B. *Frost action, in Interpretation of Micromorphological Features of Soils and Regoliths*. (Elsevier, 2010).
7. Bertran, P. & Texier, J. P. Facies and microfacies of slope deposits. *Catena* **35**, 99–121 (1999).
8. Pawelec, H. & Ludwikowska-Kędzia, M. Macro- and Micromorphologic Interpretation of Relict Periglacial Slope Deposits from the Holy Cross Mountains, Poland. *Permafr. Periglac. Process.* **27**, 229–247 (2016).

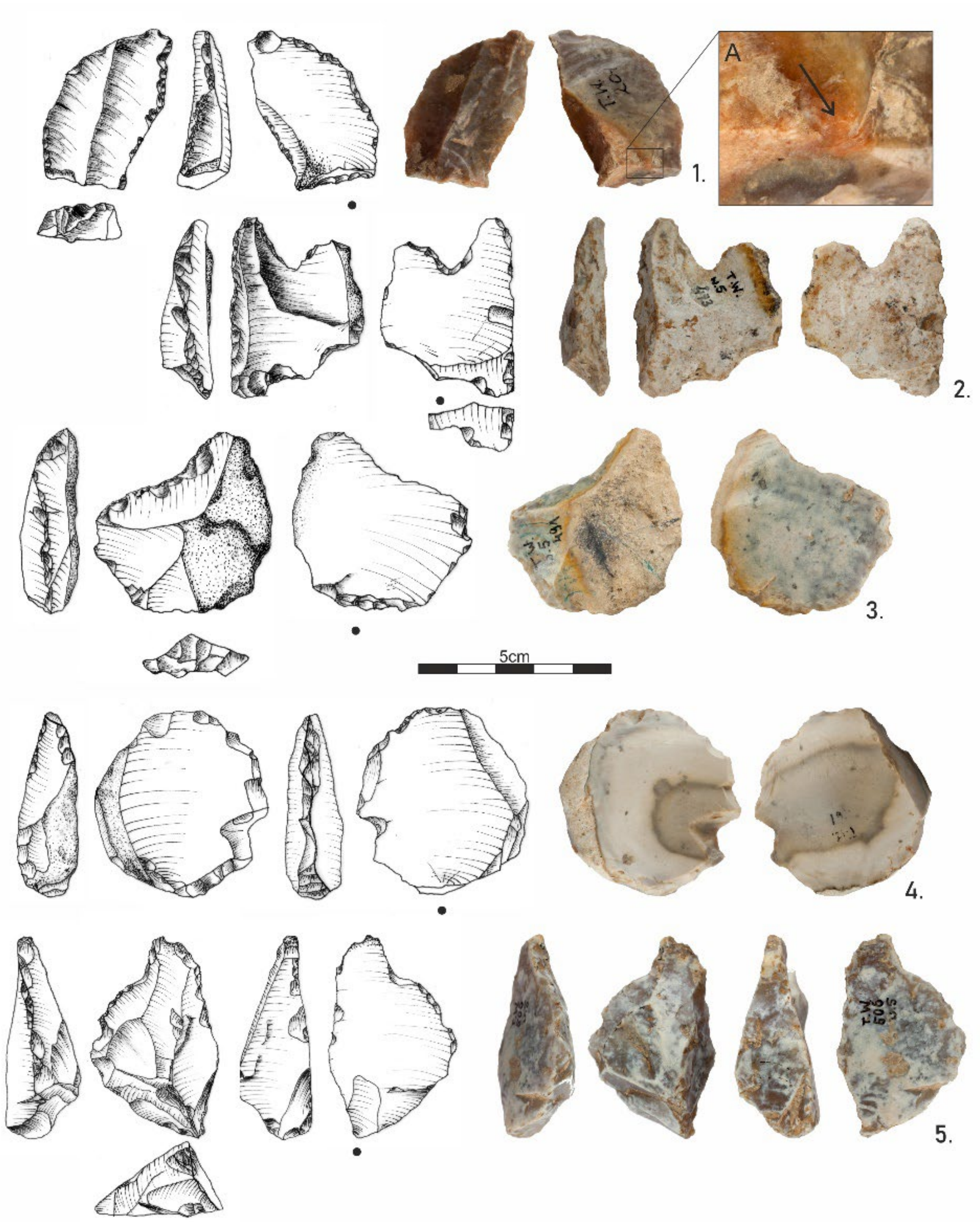

Supplement Fig.S10. *Flake debitage from Tunel Wielki Cave layer F consisting of backed flakes with a single sharp edge. One can observe heavily postdepositionaly retouched edges. A. Protruding point of percussion on the ventral side is marked with an arrow.*

Supplementary Materials Table S11

High-resulution photos of the artefacts are available: **https://doi.org/10.5281/zenodo.7047839**

Nr inw	year	No in sqm	trench	metre	layer	type of layer	depth	date	size					type of artefact	shape of artefact	cross section	profile	axis of the symmetry	whole piece	fragment	direction of scars	cortex		location of cortex	scars of unsuccesful striking in the proximal part	bulb	bulbar scar	placement of strike on the platform	distal part	shape of a butt	size of the butt		platform preparation	strikes paralell to interscar-ridges	strikes paralell to the ridges on the striking platform	line of butt	overhang platform	comment	retouch
									lenght	width	thickness in max width	max thickness	angle	flake, blade, bladelet, core, preform, chip, chunk								cortex	surface of the cortex (%)								width	thickness							
142	1967	142	I	8D	F	F	220-240	30 VI 67	39,87	24,72	11,03	11,03	n	flake	irregular	n	straight	asymetric	yes		ortagonal	no			no	no	yes	n	n	n			n	no	n	n	n	overheated	yes
161	1967	3	I	7D	F	F	240-250	1 VII 67	47,11	45,55	14,89	15,19	90	flake	oval	irregular	straight	symetric	yes		unidirectional	yes	10	lateral left	no	no	no	central	alternated platform	irregular	19,16	9,93	cortical	no	no	convex	no	used as core?	yes
179	1967	[-]	I	7D	J	lower	250-260	3 VII 67	45,17	41,26	18,16	20,76	n	core																									
188	1967	[-]	I	8D	G	lower	260-270	3 VII 67	44,87	59,43	17,12	19,05	n	flake	irregular	irregular	curved	asymetric	yes		convergent	yes	5	central	no	big	no	central	retouched	convex-convex	27,38	7,5	plan	no	no	convex	n	tool?	yes
189	1967	[-]	I	8D	G	lower	260-270	3 VII 67	35,47	21,93	10,22	10,22	67	flake	oval	planoconvex	straight	asymetric	yes		no scars	yes	100		no	no	no	n	cortex	n	n	n	cortical	n	no	n	no		yes
190	1967	[-]	I	8C	I	lower	260-270	3 VII 67	40,06	21,05	16,33	16,37	87	core	n	n	n	n	no	distal	no scars	yes	100		no	n	n	n	cortex	n	n	n	n	n	n	n	n	core on flake	yes
192	1967	[-]	I	7D	F	F	260-270	3 VII 67	22,71	26,7	6,61	7,23	n	flake	irregular	n	straight	asymetric	no	proximal	ortagonal	no			no	big	no	n	n	n	n	n	n	n	n	n	n		yes
199	1967	[-]	I	6D	F	F	260-270	3 VII 67	32,1	22,35	8,39	10,89	67	flake	oval	right angle triangular	straight	asymetric	yes		convergent	no			no	no	n	n	sharp	n	n	n	crushed	no	no	n	no	Kombewa flake/tool?	yes
204A	1967	[-]	I	7D	F	F	270-280	3 VII 67	36,69	25,76	8,35	11	65	flake	point end	right angle trapezoid	straight	asymetric	yes		biderectional	yes	5	lateral right	no	small	no	central	sharp	trapezoid	20,32	12,49	plan	interridges II	no	plan	no		yes
204B	1967	[-]	I	7D	F	F	270-280	3 VII 67	49,03	37,46	17,46	22,69	70	core																								core on flake	
206	1967	206	I	6D	F	F	270-280	3 VII 67	30,74	20,49	5,45	5,98	n	flake	rectangular	planoconvex	straight	asymetric	yes		unidirectional	no			no	no	yes	n	sharp	n	n	n	n	no	n	n	no	Kombewa flake/tool?	yes
207	1967		I	6D	F	F	270-280	3 VII 67	23,24	27,45	7,91	8,56	n	flake	n	n	n	asymetric	no	distal	unidirectional	no			n	n	n	n	hinge	n	n	n	n	no	n	n	no		yes
224	1967	[-]	I	8D	I	lower	280-290	5 VII 67	14,22	17,94	9,26	9,59	n	chunk																									
231	1967	[-]	I	7D	F	F	280-290	5 VII 67	28,41	9,16	9,09	9,35	n	flake	rectangular	trapezoid	straight	asymetric	no	longitudinal fracture	ortagonal	no			no	no	no	n	hinge	n	n	n	n	n	n	n	n		no
232	1967	[-]	I	7D	F	F	280-290	5 VII 67	10,11	15,82	5,85	5,85	n	flake	n	n	n	asymetric	no	medial	no scars	yes	90		n	n	n	n	n	n	n	n	n	n	n	n	n		no
261	1967	[-]	I	6D	J	lower	290-300	6 VII 67	16,43	26,41	4,02	5	n	flake	trapezoid divergent	right angle triangular	straight	asymetric	yes		unidirectional	no			no	no	n	n	hinge	n	n	n	crushed	no	n	n	no		no
293	1967		I	6D	J	lower	300-310	7 VII 67	43,91	35,29	13,31	18,04	65	flake	oval	triangular	straight	asymetric	yes		ortagonal	no				no	yes	central	sharp	linear	29,25	6,01	two-scars	yes	yes	plan	n		yes
296	1967		I	7D	J	lower	300-310	7 VII 67	35,16	29,92	22,29	22,78	n	chunk																								core?	
297	1967		I	7D	J	lower	300-310	7 VII 67	29,35	11,88	4,65	8,57	n	chunk																									
300	1967		I	7D	H	lower	300-310	7 VII 67	40,46	31,72	19,31	19,31	70	core																								part of the core	
345	1967		I	8D	M	lower	330-340	12 VII 67	31,66	21,3	11,99	16,21	n	chunk																									
436	1968	436	III	5E	E	erosion	210-220	20 VI 68	50,27	28,89	22,3	23,02		chunk																									
443	1968		III	6-7E	F	F	220-230	24 VI 68	29,43	31,8	16,69	16,69	n	core																									
444	1968		III	6-7E	F	F	220-230	24 VI 68	34,51	30,77	13,65	15,36	n	flake	n	n	n	n	no	distal	ortagonal	yes	60	distal	no	n	n	n	cortex	n	n	n	n	n	n	n	n	used as core?	yes
446	1968		III	6-7E	F	F	220-230	24 VI 68	62	35,11	23,45	25,77	75	core																									
457	1968		III	5E	F	F	240-250	25 VI 68	50,39	29,71	12,61	14,67	65	flake	irregular	right angle triangular	twisted	asymetric	yes		biderectional	yes	20	lateral right	no	no	yes	right	alternated platform	irregular	21,61	5,86	plan	no	no	plan	no		yes
472	1968	472	III	6E	F	F	250-260	25-26 VI 68	28,09	34,76	14,51	14,51	n	flake	triangular	triangular	straight	asymetric	yes		unidirectional	no			no	no	yes	left	sharp	triangular	25,85	12,77	uncertain	yes	no	convex	no		yes
473	1968	473	III	5E	F	F	250-260	25-26 VI 68	45,91	36,28	10,15	10,15	65	flake	irregular	irregular	straight	asymetric	yes		biderectional	yes	10	lateral right	no	big	no	central	sharp	irregular	23,12	8,63	plan	no	no	convex	no		yes
486	1968		III	5E	O	lower	260-270	25-26 VI 68	78,79	60,1	63,68	63,68	80	core																									
489	1968	489	III	5E	F	F	270-280	26 VI 68	51,07	27,92	8,97	10,1	n	flake	oval	triangular	straight	asymetric	yes		unidirectional	yes	60	lateral right	no	no	n	n	cortex	n	n	n	n	yes	n	n	n		yes
490	1968	490	III	6E	F	F	270-280	26 VI 68	73,9	57,5	9,65	9,65	75	flake	irregular	multiscar	twisted	asymetric	yes		radial	no			yes	big	n	n	sharp	n	n	n	n	yes	n	convex	no	free-hand?	yes
491	1968	491	III	6E	F	F	270-280	26 VI 68	46,22	46,14	17,6	17,6	74	flake	irregular	triangular	straight	asymetric	yes		ortagonal	yes	60	lateral right	no	no	no	central	cortex	triangular	29,91	10,67	uncertain	yes	n	plan	no		yes
493	1968	493	III	7E	F	F	270-280	26 VI 68	45,49	25,38	11,93	12,12	60	flake	oval	trapezoid	straight	asymetric	yes		unidirectional	yes	10	central	no	small	no	right	alternated platform	convex-convex	24,27	11,64	plan	yes	no	convex	n	tool?	yes
506A	1968	506	III	5E	F	F	280-290	27 VI 68	50,85	37	25,86	26,58	n	chunk																									
506B	1968	506	III	5E	F	F	280-290	27 VI 68	47,27	45,1	24,67	32,43	70	core																									
506C	1968	506	III	5E	F	F	280-290	27 VI 68	48,16	33,1	19	19	n	core	irregular	irregular	straight	asymetric	yes		unidirectional convergent	yes	5	distal	no	no	yes	n	alternated platform	n	n	n	n	yes	n	n	no	core on flake	yes
529	1968		III	5E	F	F	300-310	28 VI 68	61,48	37,61	33,1	33,1	n	chunk																								raw meterial nodule with single scars	
537	1968		III	5E	F	F	310-320	1 VII 68	26,89	25,78	11,86	11,86	75	flake	irregular	trapezoid	straight	asymetric	yes		biderectional	no			no	no	no	central	alternated platform	trapezoid	11,85	7,94	plan	yes	no	convex	no		yes
539A	1968		III	5E	F	F	320-330	2 VII 68	17,61	28,96	6,37	6,37	n	flake	n	planoconvex	straight	n	no	distal	unidirectional	no			n	n	n	n	sharp	n	n	n	n	no	n	n	n	Kombewa flake	yes
539B	1968		III	5E	F	F	320-330	2 VII 68	34,26	28,33	12,58	17,3	65	flake	n	n	straight	n	no	longitudinal fracture	ortagonal	yes	30	proximal	no	n	yes	n	n	n	n	n	n	n	n	n	n		yes
576	1968		III	5E	G+J	lower	370-380	3 VII 68	49,21	30,81	13	14,15	74	flake	irregular	trapezoid	straight	asymetric	yes		unidirectional	yes	30	lateral right + proximal	no	no	no	n	alternated platform	convex-convex	6,75	3,41	plan	yes	no	plan	no		no
138	1968	[-]	I	7D	E	erosion	220-240	30 VI 67						chunk																									
160	1968	[-]	I	7C	E	erosion	240-250	1 VII 67						core																									
608	1968		IV	1-12xd	E	erosion	40-50	8-9 VII 68						flake	n	n	straight	n	no	distal	ortagonal	no			n	n	n	n	sharp	n	n	n	n	n	n	n	n		yes
A281	2018	281	2	D9	E	erosion	170-180	1 VIII 18	19,38	18,35	8,52	9,45		flake	n	n	straight	asymetric	no	distal	ortagonal	yes	30	distal	n	n	n	n	cortex	n	n	n	n	n	n	n	n		yes
A282	2018	282	2	D9	F	F	220-240	7 VIII 18	38,17	48,36	16,09	16,09		flake	oval	trapezoid	straight	asymetric	yes		biderectional	yes	70	lateral right + proximal	no	no	no	left	alternated platform	trapezoid	45,27	18,3	cortical	no	no	convex	no		yes
TWB699	2018	699	2	E9	D	erosion	160-170	27 VII 18	26,14	15,91	6,59	6,59	n	chunk																									
TWB702	2018	702	2	E9	D	erosion	160-170	27 VII 18						chip																									
TWB704	2018	704	2	E9			160-170	27 VII 18						not artefact																									
TWB706a	2018	706	2	E9	D	erosion	160-170	27 VII 18						chip																								no damage on edges	
TWB706b	2018	706	2	E9	D	erosion	160-170	27 VII 18						chip																								ventral thinning	
TWB712	2018	712	2	D9	D	erosion	160-170	27 VII 18						chip																									
TWB727	2018	727	2	E9	mixed	mixed	170-180	1 VIII 18						chunk																									
TWB730	2018	730	2	D9	E	erosion	170-180	1 VIII 18	11,62	14,88	3,36	3,36		flake	n	n	n	n	no	medial	ortagonal	no			n	n	n	n	n	n	n	n	n	n	n	n	n		yes
TWB736	2018	736	2	E9	E	erosion	180-190	1 VIII 18						chip																									
TWB742	2018	742	2	D9	R	lower	190-200	1 VIII 18						chunk																									
TWB746a	2018	746	2	E9	mixed	mixed	190-200	1 VIII 18						chip																									
TWB746b	2018	746	2	E9	mixed	mixed	190-200	1 VIII 18						chunk																									
TWB747	2018	747	2	E9	E	erosion	190-200	1 VIII 18						chip																								no damage on edges	
TWB749a	2018	749	2	D9	F	F	200-220	2 VIII 18						chip																									
TWB749b	2018	749	2	D9	F	F	200-220	2 VIII 18						chip																									
TWB749c	2018	749	2	D9	F	F	200-220	2 VIII 18						chip																									
TWB749d	2018	749	2	D9	F	F	200-220	2 VIII 18						chip																									
TWB749e	2018	749	2	D9	F	F	200-220	2 VIII 18						chip																									
TWB749f	2018	749	2	D9	F	F	200-220	2 VIII 18						chip																								fragment	
TWB749g	2018	749	2	D9	F	F	200-220	2 VIII 18						chip																									
TWB749h	2018	749	2	D9	F	F	200-220	2 VIII 18						chip																									
TWB749i	2018	749	2	D9	F	F	200-220	2 VIII 18						chip																									
TWB750	2018	750	2	D9	E	erosion	200-220	2 VIII 18	16,96	22,06	7,05	7,05	n	flake	oval	planoconvex	straight	asymetric	yes		no scars	yes	100		no	no	no	central	alternated platform	planoconvex	16,94	5,72	cortical	no	no	plan	no		no
TWB756	2018	756	2	E9	E	erosion	200-220	2 VIII 18						chip																									
TWB760a	2018	760	2	D9	H	lower	200-220	2 VIII 18						chunk																									
TWB760b	2018	760	2	D9	H	lower	200-220	2 VIII 18						chip																									
TWB771	2018	771	2	E9	J1	lower	220-240	7 VIII 18						chip																									
TWB773	2018	773	2	D9	G	lower	220-240	7 VIII 18						chip																									
TWB774	2018	774	2	D9	H	lower	220-240	7 VIII 18	17,3	16,27	4,22	4,22	n	flake	n	n	n	asymetric	no	medial	no scars	no			n	n	n	n	n	n	n	n	n	n	n	n	n	termic changes	no
TWB780	2018	780	2	E9	I	lower	220-240	8 VIII 18	7,93	9,55	2,09	2,09	n	flake	n	trapezoid	straight	n	no	medial	unidirectional	no			n	n	n	n	n	n	n	n	n	n	n	n	n		yes
TWB781a	2018	781	2	E9	K2	erosion	220-240	8 VIII 18						chip																									
TWB781b	2018	781	2	E9	K2	erosion	220-240	8 VIII 18						chunk																									
TWB783a	2018	783	2	E9	J1	lower	220-240	8 VIII 18						chip																									
TWB783b	2018	783	2	E9	J1	lower	220-240	8 VIII 18						chunk																									
TWB783c	2018	783	2	E9	J1	lower	220-240	8 VIII 18						chip																									
TWB783d	2018	783	2	E9	J1	lower	220-240	8 VIII 18						chip																									
TWB785a	2018	785	2	D9	F	F	200-240	8 VIII 18						chip																								cortical	
TWB785b	2018	785	2	D9	F	F	200-240	8 VIII 18						chip																									
TWB785c	2018	785	2	D9	F	F	200-240	8 VIII 18						chip																									
TWB787a	2018	787	2	E9	K2	erosion	220-230	8 VIII 18						chip																									
TWB787b	2018	787	2	E9	K2	erosion	220-230	8 VIII 18						chip																									
TWB787c	2018	787	2	E9	K2	erosion	220-230	8 VIII 18						chunk																									
TWB788	2018	788	2	E9	L1	erosion	230-240	8 VIII 18						chip																									
TWB789a	2018	789	2	E9	K2	erosion	230-240	8 VIII 18						chip																									
TWB789b	2018	789	2	E9	K2	erosion	230-240	8 VIII 18						chip																									
TWB789c	2018	789	2	E9	K2	erosion	230-240	8 VIII 18						chip																									
TWB789d	2018	789	2	E9	K2	erosion	230-240	8 VIII 18						chip																									
TWB789e	2018	789	2	E9	K2	erosion	230-240	8 VIII 18						chip																									
TWB789f	2018	789	2	E9	K2	erosion	230-240	8 VIII 18						chip																									
TWB789g	2018	789	2	E9	K2	erosion	230-240	8 VIII 18	5,29	4,11	0,98	0,98		flake	n	n	n	n	no	medial	n	no			n	n	n	n	n	n	n	n	n	n	n	n	n		no
TWB789h	2018	789	2	E9	K2	erosion	230-240	8 VIII 18						chip																									
TWB789i	2018	789	2	E9	K2	erosion	230-240	8 VIII 18						chip																									
TWB793a	2018	793	2	D9	I	lower	230-240	8 VIII 18						chip																									
TWB793b	2018	793	2	D9	I	lower	230-240	8 VIII 18						chunk																									
TWB793c	2018	793	2	D9	I	lower	230-240	8 VIII 18						chip																									
TWB793d	2018	793	2	D9	I	lower	230-240	8 VIII 18						chip																									
TWB796a	2018	796	2	E9	K2	erosion	230-240	9 VIII 18						chip																									
TWB796b	2018	796	2	E9	K2	erosion	230-240	9 VIII 18						chip																									
TWB796c	2018	796	2	E9	K2	erosion	230-240	9 VIII 18						chip																									
TWB796d	2018	796	2	E9	K2	erosion	230-240	9 VIII 18						chip																									
TWB796e	2018	796	2	E9	K2	erosion	230-240	9 VIII 18						chip																									
TWB796f	2018	796	2	E9	K2	erosion	230-240	9 VIII 18						chip																									
TWB830	2018	830	2	E9	K2	erosion	240-260	10 VIII 18						chip																									
TWB834a	2018	834	2	E9	K2	erosion	240-260	10 VIII 18						chip																									
TWB834b	2018	834	2	E9	K2	erosion	240-260	10 VIII 18						chip																									
TWB837a	2018	837	2	E9	K2	erosion	240-260	10 VIII 18						chip																									
TWB837b	2018	837	2	E9	K2	erosion	240-260	10 VIII 18						chip																									
TWB837c	2018	837	2	E9	K2	erosion	240-260	10 VIII 18						chip																									
TWB840a	2018	840	2	E9	K2	erosion	240-260	10 VIII 18						chip																									
TWB840b	2018	840	2	E9	K2	erosion	240-260	10 VIII 18						chip																									
TWB842	2018	842	2	D9	J2	lower	260-280	10 VIII 18						chunk																									
TWB845	2018	845	2	E9	K2	erosion	260-280	10 VIII 18						chunk																									
TWB846	2018	846	2	E9	L1	erosion	260-280	10 VIII 18						chunk																									
TWB847	2018	847	2	E9	L2	erosion	260-280	10 VIII 18						chip																									
TWB848	2018	848	2	E9	L1	erosion	260-280	10 VIII 18						chip																									
TWB851a	2018	851	2	E9	L1	erosion	260-280	10 VIII 18						chip																									
TWB851b	2018	851	2	E9	L1	erosion	260-280	10 VIII 18						chip																									
TWB852	2018	852	2	E9	L	erosion	260-280	10 VIII 18						chip																									
TWB854a	2018	854	2	D9	K1	lower	280-300	10 VIII 18						chunk																									
TWB854b	2018	854	2	D9	K1	lower	280-300	13 VIII 18						chunk																									
TWB856a	2018	856	2	E9	M1	lower	280-300	13 VIII 18						chip																									
TWB856b	2018	856	2	E9	M1	lower	280-300	13 VIII 18						chunk																									
TWB857	2018	857	2	D9	K1	lower	280-300	13 VIII 18						chunk																									
TWB857	2018	857	2	D9	K1	lower	280-300	13 VIII 18						chip																									
TWB860a	2018	860	2	E9	L2	erosion	280-300	13 VIII 18						chip																								no damage on edges	
TWB860b	2018	860	2	E9	L2	erosion	280-300	13 VIII 18						chunk																								no damage on edges	
TWB861a	2018	861	2	E9	L2	erosion	280-300	13 VIII 18						chip																									
TWB861b	2018	861	2	E9	L2	erosion	280-300	13 VIII 18						chunk																									
TWB863a	2018	863	2	E9	M	lower	280-300	13 VIII 18						chip																									
TWB863b	2018	863	2	E9	M	lower	280-300	13 VIII 18						chip																									
TWB863c	2018	863	2	E9	M	lower	280-300	13 VIII 18						chip																									
TWB863d	2018	863	2	E9	M	lower	280-300	13 VIII 18						chip																									
TWB863e	2018	863	2	E9	M	lower	280-300	13 VIII 18						chunk																									
TWB863f	2018	863	2	E9	M	lower	280-300	13 VIII 18						chunk																									
TWB865	2018	865	2	D9	L2	erosion	300-320	13 VIII 18						chunk																									
TWB869a	2018	869	2	E9	M1	lower	300-320	13 VIII 18						chip																									
TWB869b	2018	869	2	E9	M1	lower	300-320	13 VIII 18						chunk																									
TWB870	2018	870	2	E9	M1	lower	300-320	13 VIII 18						chip																									
TWB874a	2018	874	2	E9	M2	lower	300-320	13 VIII 18						chip																									
TWB874b	2018	874	2	E9	M2	lower	300-320	13 VIII 18						chip																									
TWB874c	2018	874	2	E9	M2	lower	300-320	13 VIII 18						chip																								termic changes	
TWB874d	2018	874	2	E9	M2	lower	300-320	13 VIII 18						chunk																									
TWB876a	2018	876	2	E9	M2	lower	300-320	13 VIII 18						chip																									
TWB876b	2018	876	2	E9	M2	lower	300-320	13 VIII 18						chunk																									
TWB879	2018	879	2	D9	M1	lower	300-320	13 VIII 18						chunk																									
TWB880	2018	880	2	E9	N	lower	300-320	13 VIII 18						chip																									
TWB889	2018	889	2	E9	mixed	mixed	aning secti	14 VIII 18						chip																									
TWB893	2018	893	2	D9	mixed	mixed	aning secti	14 VIII 18						chip																									
TWB896a	2018	896	2	D9	M2	lower	320-340	14 VIII 18						chip																									
TWB896b	2018	896	2	D9	M2	lower	320-340	14 VIII 18						chunk																									
TWB897	2018	897	2	D9	M2	lower	320-340	14 VIII 18						chip																									
TWB898	2018	898	2	E9			320-340	14 VIII 18						not artefact																									
TWB899	2018	899	2	E9	N	lower	320-340	14 VIII 18						chip																									
TWB900a	2018	900	2	E9	N	lower	320-340	14 VIII 18						chip																									
TWB900b	2018	900	2	E9	N	lower	320-340	14 VIII 18						chunk																									
TWB902	2018	902	2	E9	N	lower	320-340	14 VIII 18						chunk																									
TWB903	2018	903	2	E9	N	lower	320-340	14 VIII 18						chunk																									
TWB907	2018	907	2	D9,E9	O	lower	340-360	14 VIII 18						chip																									
TWB114	2018	114	1	-	mixed	mixed	30-50	13 VII 18	7,13	14,86	0,95	0,95	n	flake	n	n	n	n	no	medial	n	no			n	n	n	n	n	n	n	n	n	n	n	n	n		yes
TWB199	2018	199	1	-	mixed	mixed	0-90	13 VII 18						chip																									
TWB200	2018	200	1	-	mixed	mixed	0-90	13 VII 18						chunk																									
TWB316	2018	316	1	-	mixed	mixed	90-120	14 VII 18	20,5	18,3	8	8,38	n	flake	n	triangular	straight	n	no	distal	biderectional	yes	40	distal	n	n	n	n	sharp	n	n	n	n	n	n	n	n		yes
TWB353	2018	353	1	-	mixed	mixed	120-140	14 VII 18						chunk																									
TWB543	2018	543	1	-	mixed	mixed	160-180	25 VII 18	48,08	21,61	13,31	14,97		flake	rectangular	right angle triangular	straight	asymetric	yes		unidirectional	yes	70	lateral right	no	no	yes	right	hinge	triangular	14,42	4,96	plan	yes	no	convex	no		yes
TWB805	2018	805	1	-	mixed	mixed	300-320	9 VIII 18						chip																									
TWB813	2018	813	1	-	mixed	mixed	300-320	9 VIII 18						chip																									
TWB888	2018	888	1+2	-	mixed	mixed	-	14 VIII 18						chip																									

	chip	flake	core	chunk	chunk/core	
erosion	40	5	1	10	0	56
F	12	21	5	2	0	40
lower	27	7	4	23	0	61
mixed	7	3	0	4	0	14
	86	36	10	39	0	171

Lithological s	Layer	flake	core	chip	chunk	TOTAL
Series III	D	0	0	4	1	5
	E	4	1	3	2	10
	K2	1	0	25	3	29
	L1	0	0	4	1	5
	L2	0	0	3	3	6
	L	0	0	1	0	1
Series II	F	21	5	12	2	40
	G	2	0	1	0	3
	G+J	1	0	0	0	1
	H	1	1	1	1	4
	I	1	1	3	2	7
	J	2	1	0	2	5
	J1	0	0	4	1	5
	J2	0	0	0	1	1
	K1	0	0	1	3	4
	M1	0	0	3	3	6
	M2	0	0	6	3	9
	M	0	0	4	3	7
	N	0	0	3	3	6
	O	0	1	1	0	2
	R	0	0	0	1	1
	mixed	3	0	7	4	14
TOTAL		36	10	86	39	171

chip	86
flake	36
core	10
chunk	39
chunk/cor	0
	171

leaf	0	right angle triangular	4
oval	8	triangular	5
rectangular	3	trapezoid	6
triangular	1	right angle trapezoid	1
point end	1	multiscar	1
circular	0	planoconvex	4
trapezoid	0	waved	0
convergent	0	irregular	4
trapezoid divergent	1	n	13
irregular	10		
n	14		
	38		38

	2018	1967	1968		1967/68
chip	86	0	0	chip	0
flake	10	13	13	flake	26
chunk	31	4	4	chunk	8

unidirectional	11	mean	45,25
biderectional	6	no	18
multidirectional	0		
radial	1		
convergent	1		
unidirectional conver	2		
no scars	5		
bidirectional 1/3	0		
ortagonal	10		
	36		

		lip	0
		big	4
lateral right	6	medium	0
lateral left	1	small	2
distal	3	no	18
proximal	0	big+lip	0
other	0	small + lip	0
lateral rights +	0	medium + lip	0
lateral left + di	1	n	14
lateral right + p	3		38
lateral left + pr	0		
central	2		
	34		

n	17
yes	8
no	13
	38

	22,579	8,987857	
circular	0	plan (odnov	0
triangular	3	two-scars	1
trapezoid	3	multiscars	0
planoconvex	1	facetted	0
convex-conv	3	cortical	4
linear	1	crushed	2
chapeaux de	0	uncertain	2
point	0	n	21
irregular	3		30
n	24		
	38		

plan	6
convex	9
concave	0
n	23

Supplement Table S12 *Distribution of artefacts within Pleistocene layers of Tunel Wielki Cave.*

Lithological series	*Layer*	*flake*	*core*	*chip*	*chunk*	***TOTAL***
Series III	*D*	0	0	4	1	**5**
	E	4	1	3	2	**10**
	K2	1	0	25	3	**29**
	L1	0	0	4	1	**5**
	L2	0	0	3	3	**6**
	L1+L2	0	0	1	0	**1**
Series II	*F*	21	5	12	2	**40**
	G	2	0	1	0	**3**
	G+J	1	0	0	0	**1**
	H	1	1	1	1	**4**
	I	1	1	3	2	**7**
	J1+ J2	2	1	0	2	**5**
	J1	0	0	4	1	**5**
	J2	0	0	0	1	**1**
	K1	0	0	1	3	**4**
	M1	0	0	3	3	**6**
	M2	0	0	6	3	**9**
	M1+ M2	0	0	4	3	**7**
	N	0	0	3	3	**6**
	O	0	1	1	0	**2**
	R	0	0	0	1	**1**
	mixed	3	0	7	4	**14**
**TOTAL**		**36**	**10**	**86**	**39**	**171**

Supplement Table S13 *Comparison of the mean length of artefacts from Tunel Wielki Cave and other Lower Palaeolithic assemblages after Burdukiewicz*[1] *.*

	mean artefacts lenght (mm)
**Tunel Wielki Cave**	**40**
Xiaochangling	27
Donggutuo	30
Kuldara	30
Ruhama	19
Isernia	23
Cinfonare	16
Vértesszölös	18
Bilzingsleben	16
Rusko 33	16
Rusko 42	17
Trzebnica 2	20

References

1. Burdukiewicz, J. M. Lower Palaeolithic microlithic technology in Central Europe and wooden tools. in *Les premiers peuplements en Europe: Colloque international : Données récentes sur les modalités du peuplement sur le cadre chronostratigraphique, géologique et paléogeographique des industries du Paléolithique ancien et moyen en Europe* (eds. Molines, N., Moncel, M.-H. & Monnier, J.-L.) 341–368 (British Archaeological Reports, 2005).

Supplement Data 14

Bird assemblage analysis

Material and methods

Bird bones at Tunel Wielki Cave were excavated during the old (Chmielewski's) excavation as well as during the newest one. The former assemblage was already studied by Bocheński[1,2] and all the bones were ascribed to Holocene. This stratigraphy has been verified, and the bones rendered as Pleistocene's were reexamined. The bones from the newest excavations were identified by K. Wertz and Teresa Tomek; the bone surface was checked for possible human, animal, and environment modifications (see e.g.[3,4]). For bone identification, the collection of ISEA PAS was used along with the bird bone identifications manuals[5]. In this study, only the bones attributed to Pleistocene layers are included. All bird bones from Tunel Wielki are stored at ISEA, PAS.

Results and comments

Bird bones from the Pleistocene layers at Tunel Wielki are very scarce (Table below).

Number of identified bird remains (NISP) from the Pleistocene's layer at Tunel Wielki Cave.

Layer	J2	K1	K2
Gallus gallus	1*		
Galliformes indet.		1	
Periparus ater			1

* - probably modern admixture

Detailed description of the bird bones:

Coal Tit (*Periparus ater*): its homogeneously black-stained bone (tibiotarsus) comes from the new excavations. The bone was found in layer K2, among the Middle Pleistocene sediment fouled by the Late Pleistocene impurities resulting from the erosion episode. Consequently, the bone might have been deposited as early as during MIS13-11, but its deposition during MIS3 is also possible. Tunel Wielki is the third archaeological site where the remains of Coal Tit were

unearthed; the former two are Obłazowa Cave (the Late Glacial layer, MIS 1) and Rockshelter in Krucza Skała (the Holocene layer)[6]. Coal Tit is a forest species; it occupies either coniferous forests, often with spruce, or mixed forests with birch, pine, or larch[7].

Galliformes indet.: the bone fragment (sternum) was primarily attributed to the Holocene and identified as domestic chicken (*Gallus gallus*)[2]. Verified stratigraphy (Layer K1) demonstrates the bone is much older (the Pleistocene, MIS 13-11). The bone is black-stained, heavy, and probably petrified. Taxonomic re-examination of the bone did not confirm its former taxonomic identification. The bone surely comes from a middle-sized galliform (approximately the size of a Black Grouse male or Capercaillie female) but it bears a mosaic of minute anatomic details which differentiates it from each species in the comparative collection. It may be a noteworthy coincidence that the period of the bone deposition (MIS 13-11, MNQ 23) is adjacent to the period (MNQ 22) in which the modern forms of such tetraonids as the Capercaillie and the Black Grouse are thought to have developed from their ancestors[8].

Domestic chicken (*Gallus gallus*): the bone (femur) was among the remains identified by Z. Bocheński but it was not included in either of the papers (i.e.[1,2]). The attached stratigraphic data, after revision, attribute the bone to the Pleistocene Layer J2, MIS 13-11. The re-examination of the bone confirmed it belongs to domestic chicken. The bone wears distinct traces of gnawing (very well preserved), its medullar cavity is filled with medullary bone, and the bone has creamy white color, similar to the bones in the Holocene's assemblage. Good state of the bone preservation, its taxonomic affiliation, and the fact that several chicken bones were found in the Holocene sediment, strongly suggest the bone is the Holocene's admixture.

References

1. Bocheński, Z. *The birds of the Late Quaternary of Poland (Ptaki młodszego czwartorzędu Polski)*. (Państwowe Wydawnictwo Naukowe, 1974).
2. Bocheński, Z. Kopalne ptaki z jaskiń i schronisk Doliny Sąspowskiej. in *Jaskinie Doliny Sąspowskiej - Tło przyrodnicze osadnictwa pradziejowego* (ed. Chmielewski, W.) 47–77 (Prace Instytutu Archeologii UW, Wydawnictwo Uniwersytetu Warszawskiego, 1988).
3. Fernández-Jalvo, Y. & Andrews, P. Atlas of taphonomic identifications. in *Vertebrate Paleobiology and Paleoanthropology* (Springer, 2016). doi:10.1007/978-94-017-7432-1.

4. Laroulandie, V. Anthropogenic versus non-anthropogenic bird bone assemblages: New criteria for their distinction. in *Biosphere to lithosphere: new studies in vertebrate taphonomy. Proceedings of the 9th conference of ICAZ, Durham, 23-28 august 2002* (ed. O'Connor, T.) 25–30 (Oxbow Books, 2005).

5. Erbersdobler, K. Vergleichend morphologische Untersuchungen an Einzelknochen des postcranialen Skeletts in Mitteleuropa vorkommender mittelgrober Hühnervögel. (Ludwig-Maximilians-Universitöt München, Munich, 1968).

6. Bocheński, Z., Bocheński, Z. M. & Tomek, T. *A History of Polish Birds*. (Institute of Systematics and Evolution of Animals (Polish Academy of Sciences), 2012).

7. Voous, K. H. *Atlas of European Birds*. (Nelson, 1960).

8. Mourer-Chauviré, C. The Pleistocene avifaunas of Europe. *Archaeofauna* **2**, 53–66 (1993).

Supplement Data 15

Mollusc analysis

Only four mollusc taxa represented by 4 individuals were noted in the Middle Pleistocene deposits of Tunel Wielki Cave. *Isognomostoma isognomostomos* (Schröter) is a shade-loving species usually inhabiting the humid mountain forests, whereas *Clausilia dubia* Draparnaud is a mesophilous snail typical of humid shady rocks[1,2]. The assemblage is completed by a single shell fragment of *Cepaea* sp. and the apex of Clausiliidae snail (Table below).

The very scarce shell material excludes any palaeonvironmental reconstructions for the Middle Pleistocene in Tunel Wielki based on malacological record. However, the presence of *I. isognomostomos* (if not reworked from the younger deposits) in layer G/H implies rather warm and humid climate conditions and forest environment.

*Molluscs from the Middle Pleistocene deposits of Tunel Wielki Cave. Ecological groups (after Alexandrowicz and Alexandrowicz[3]): F – shade-loving taxa, M – mesophilous taxa; f – shell fragments, * – crushed shell.*

E	Taxon	Layer			
		G/H	I	J2	M2
F	*Isognomostoma isognomostomos* (Schröter)	1*			
M	*Clausilia dubia* Draparnaud		1		
	Cepaea sp.			1f	
	Clausiliidae				1

References

1. Wiktor, A. *Ślimaki lądowe Polski*. (Wydawnictwo Mantis, 2004).
2. Welter-Schultes, F. *European non-marine molluscs, a guide for species identification*. (Planet Poster Editions, 2012).
3. Alexandrowicz, S. W. & Alexandrowicz, W. P. Analiza malakologiczna metody badań i interpretacji. (Malacological analyses methods of investigation and interpretation). *Rozpr. Wydz. Przyr. Pol. Akad. Umiejętności* **3**, 5–308 (2011).

Supplement Table S16

Large mammals distribution within layers

Taxon	Layers																																																																					Total
	Mould				Series V					Series IV		Series III													Series II																																													
	h-1	h-2	h-3	h-4	A1	A2	B	B1	B2	C	D	D/E	D/G	E	E/J1	E/I	E/F	K1/L1	K1/L2	L1	L1/L2	L2	L2/M1	L1/M	F	F+I.	E/G/H	G	G?	G/H	G/J	G+H+J	G+J	H	I	I?	I/J/H	H+I+J+L	J	J?	I/J	I+J	J+L	I/J1	J1	J1/J2	J2	J1/K1	J+K	K1	K+L	K+M	L+M	J+K+M	I+K+M	K+L+M	top M	M	M1	L2/M2	M2	M2/N	M+N	N	N+O	M+N+O	L+M+N+O	O	O/R	R	
	NISP/MNI																																																																						
Cervus elaphus (Linnaeus, 1758)	1	6/1.				5/1.		1																	2/1.	2/1.									4/1.										1							1																1			24/10.
Cervalces latifrons (Johnson, 1874)																																																																				1			1
Rangifer tarandus (Linnaeus, 1758)														1																																															1			1								3/3.
Capreolus capreolus (Linnaeus, 1758)		2/1.																																																1																					3/2.
Capreolus priscus (Soergel, 1914)																												1											1														1																		3/3.
Cervidae/Bovidae						7									1																														1		1																								10
Bison priscus (Bojanus, 1827)														1																																																									1
Bos primigenius (Bojanus, 1827)						21/1.	1																					1							1																																					24/4.
Bos taurus taurus (Linnaeus, 1758)						1																																																																		1
Bos sp. (Linnaeus, 1758)					1	8																			1			2							1																																	2			15
Ovis sp./*Capra* sp. (Linnaeus, 1758)		4				1																																																																		5
Sus scrofa (Linnaeus, 1758) /*Sus scrofa f. domestica* (Linnaeus, 1758)		11/1.				10/1.			1																																																															22/3.
Artiodactyla ident.		14			1	7								1											2										6																																	1			32
Equus ferus (Boddaert, 1785)						9/1.											1								3/1.									2/1.	1				1																																	17/6.
Coelodonta antiquitatis (Blumenbach, 1807)														2/1.											1																																															3/2.
Ungulata ident.						10																																																																		10
Ursus arctos (Linnaeus, 1758)		2/1.																																																																						2/1.
Ursus cf. arctos (Linnaeus, 1758)														1											1			1						2/1.					2/1.											1		1									1					1		3/1.			14/10.
Ursus cf. arctos (Linnaeus, 1758)/*thibetanus* (Cuvier, 1823)																																																																				1			1
Ursus deningeri (von Reichenau, 1904)		98/6.	1			4/1.				1		1		7/1.					1	1		5/2.			22/2.			9/2.	1		3/1.		1	26/2.	27/4.	3/1.		4/1.	107/5.	9/2.	1	1			2/1.		11/1.		6/1.	60/6.	12/2.	29/3.		1				48/4.	5/1.	1	4/1.	1	9/1.	14/1.	4/1.	3/1.	5/1.	52/5.		1	601/73.
Ursus sp. (Linnaeus, 1758)	8	145				1								5											7			6	2			1		2	12	2	1		41	2			2		3		2		2	12	3	1	8			1		4			1	1	1	3	5	2	1	5	1	5	298
Canis mosbachensis (Soergel, 1925)		4/1.																																					1																																	5/2.
Canis sp. (Linnaeus, 1758)																																			1			1	3											2				1				1									1	1			11
Cuon priscus (Thenius, 1954)														1																															1																										2/2.
Cuon sp. (Hodgson, 1838)/*Canis* sp. (Linnaeus, 1758)	1	5																																																																					6
Lycaon lycaonoides (Kretzoi, 1938)		1																																	2/1.												1																						1			5/4.
Vulpes vulpes (Linnaeus, 1758)		1																																																																						1
Vulpes lagopus (Linnaeus, 1758)		1																																																																						1
Vulpes sp. (Frisch, 1775)		3																																																																						3
Crocuta crocuta ssp. (Erxleben, 1777)		1																																																								1														2/2.
Panthera gombaszoegensis (Kretzoi, 1938)		1																							1			1							3/1.				5/1.	1										1																					13/7.
Panthera spelaea fossilis (von Reichenau, 1906)		2/1.												1						1					2/1.						1			4/1.	2/1.			1	12/2.						1					13/1.	1	1	2/1.		1	1		1						1				2/1.			50/20.
Homotherium aff. latidens (Owen, 1846)																																																															1								1
Gulo gulo (Linnaeus, 1758)		1																																																											1										2/2.
Meles meles ssp. (Linnaeus, 1758)											1														1																																														2/2.
Mustela putorius (Linnaeus, 1758)		1																																																																						1
Mustela nivalis (Linnaeus, 1758)																																															1																								1
Martes cf. martes (Linnaeus, 1758)		1																																											1																											2/2.
Carnivora ident.		4																																											1		4			1																					10
Unidentifiable	150	11003	1	2	31	396		2		1	3	4	1	5	1	1		1		6	1	10	2	1	11		1	4		1				4	21									1	121	1	32	1		69							2		11		16	2		15				19			11954
Total	160	11311	2	2	33	480	1	3	1	2	4	6	1	24	2	1	1	1	1	8	1	15	2	1	54	2	1	25	3	1	4	1	1	40	81	5	1	6	173	12	1	1	2	1	132	1	52	1	8	160	16	33	11	2	1	2	2	55	16	1	24	4	11	34	9	6	7	89	1	6	13162
